# Cytoprotective Effects of Dinitrosyl Iron Complexes on Viability of Human Fibroblasts and Cardiomyocytes

**DOI:** 10.3389/fphar.2019.01277

**Published:** 2019-11-11

**Authors:** Natalia Pavlovna Akentieva, Natalia Alekseevna Sanina, Artur Rasimovich Gizatullin, Natalia Ivanovna Shkondina, Tatyana Romanovna Prikhodchenko, Stanislav Ivanovich Shram, Nikolai Zhelev, Sergei Michailovich Aldoshin

**Affiliations:** ^1^Laboratory Biochemical and Cellular Studies, Department of Kinetics of Chemical and Biological Processes, Institute of Problems of Chemical Physics, Russian Academy of Sciences, Chernogolovka, Russia; ^2^Laboratory of Toxicology and Experimental Chemotherapy, Moscow State Regional University, Moscow, Russia; ^3^Faculty of Medicine, Karabük University, Karabük, Turkey; ^4^Laboratory of Structural Chemistry, Department of Structure of Matter, Institute of Problems of Chemical Physics, Russian Academy of Sciences, Chernogolovka, Russia; ^5^Faculty of fundamental physical and chemical engineering, Lomonosov Moscow State University, Moscow, Russia; ^6^Neuropharmacology Sector, Institute of Molecular Genetics, Russian Academy of Sciences, Moscow, Russia; ^7^School of Medicine, University of Dundee, Dundee, United Kingdom; ^8^Medical University Plovdiv, Plovdiv, Bulgaria

**Keywords:** dinitrosyl iron complexes, donors nitric oxide, heart disease, cell viability, membrane potential

## Abstract

Nitric oxide (NO) is an important signaling molecule that plays a key role in maintaining vascular homeostasis. Dinitrosyl iron complexes (DNICs) generating NO are widely used to treat cardiovascular diseases. However, the involvement of DNICs in the metabolic processes of the cell, their protective properties in doxorubicin-induced toxicity remain to be clarified. Here, we found that novel class of mononuclear DNICs with functional sulfur-containing ligands enhanced the cell viability of human lung fibroblasts and rat cardiomyocytes. Moreover, DNICs demonstrated remarkable protection against doxorubicin-induced toxicity in fibroblasts and in rat cardiomyocytes (H9c2 cells). Data revealed that the DNICs compounds modulate the mitochondria function by decreasing the mitochondrial membrane potential (ΔΨ_m_). Results of flow cytometry showed that DNICs were not affected the proliferation, growth of fibroblasts. In addition, this study showed that DNICs did not affect glutathione levels and the formation of reactive oxygen species in cells. Moreover, results indicated that DNICs maintained the ATP equilibrium in cells. Taken together, these findings show that DNICs have protective properties *in vitro.* It was further suggested that DNICs may be uncouplers of oxidative phosphorylation in mitochondria and protective mechanism is mainly provided by the leakage of excess charge through the mitochondrial membrane. It is assumed that the DNICs have the therapeutic potential for treating cardiovascular diseases and for decreasing of chemotherapy-induced cardiotoxicity in cancer survivors.

## Introduction

Cardiovascular disease (CVD) is the leading cause of death worldwide. According to the World Health Organization (WHO), CVD are in first place in terms of mortality in the world (WHO newsletter, 2015). Cardiovascular death and disability are a significant burden in many economies, and despite declining mortality over the past two decades, economic losses due to cardiovascular disease have risen in low- and middle-income countries (Collins et al., 2017). WHO estimates that 17.9 million people die from CVD each year, accounting for 31% of all deaths in the world (WHO newsletter, 2015; Collins et al., 2017). In particular, hypertension is a serious disease that significantly increases the likelihood of a heart attack, the risk of stroke, the development of renal failure, glaucoma, and blindness. Hypertension is one of the leading causes of premature death worldwide (WHO, 2013a). In addition, according to WHO statistics, 7.4 million people died from coronary heart disease and 6.7 million people from stroke (WHO, 2013a; WHO, 2013b). The pathology of the cardiovascular system is an extremely urgent problem, since it determines more than half of the cases of disability and adult mortality (WHO, 2013a; WHO, 2013b; WHO newsletter, 2015; Collins et al., 2017). In addition, cardiotoxicity due to chemotherapy is one of the most significant side effects in the treatment of cancer and causes significant mortality among cancer survivors. Oncological diseases are in second place after CVD by mortality (Zaorsky et al., 2017). Cancer causes a significant proportion of deaths worldwide and in particular in the United States of America and Russia (Siegel et al., 2014; Zaorsky et al., 2017). According to statistics more than 8 million people died from cancer in 2013. Cancer mortality has moved from the third leading cause of death in 1990 to the second leading cause of death in 2013, following heart disease (Coleman et al., 2003). It should be noted that modern antitumor therapy, including chemotherapy, radiation therapy, and targeted therapy, save life and allow social adaptation of cancer patients for decades. However, in the treatment of cancer there is an effect on the heart tissue that leads to a very serious complication—toxic cardiomyopathy (Łacko et al., 2002). Among the side effects of chemotherapeutic agents on the cardiovascular system, the most common and serious is heart failure with systolic ventricular dysfunction. In addition other toxic effects include myocardial ischemia, systolic or diastolic myocardial dysfunction, cardiomyopathy, chronic heart failure, arterial or pulmonary hypertension, strokes, pericarditis, arrhythmias, thromboembolic disease, and others, extremely adversely affecting the prognosis of these patients (Łacko et al., 2002; Adão et al., 2013; Truong et al., 2014). For several decades, cardiomyopathy caused by chemotherapy for cancer was mainly due to the use of cumulative doses of anthracyclines (doxorubicin, epirubicinum), and trastuzumab, which are the chemotherapeutic agents with the most pronounced cardiac side effects (Łacko et al., 2002; Adão et al., 2013; Truong et al., 2014; Zaorsky et al., 2017; Carrillo-Larco and Bernabe-Ortiz, 2018; Fidale et al., 2018; Ng et al., 2018).

Studies in the field of molecular cardiology have established the central role of nitric oxide (NO) in the regulation of vascular tone of the cardiovascular system and myocardial metabolism (Murad, 1999; Jose et al., 2013). NO is a gaseous lipophilic molecule generated in cells by three different isoforms of nitric oxide synthases (NOS): neuronal (nNOS), inducible (iNOS) and endothelial NOS (eNOS) (Shibata et al., 2013). The NO molecule is a signal bioregulator that affects various physiological and pathological processes in the cell (Chatterjee et al., 2008; Shibata et al., 2013).

NO plays an important role in protecting the body from the onset and development of cardiovascular diseases. The NO molecule is involved in the regulation of the tone of small and medium blood vessels, vasodilation, promotes relaxation of smooth muscles, exhibits anticoagulant properties, suppresses adhesion of monocytes and platelets and production of vasoconstrictors, inhibits oxidation of low-density lipoproteins and synthesis of cytokines, and affects immune response and neurotransmission (Naseem, 2005; Park et al., 2019; Raffetto et al., 2019; Tegeder, 2019). The concentration of NO is the key factor that determines its biological effect (Wink and Mitchell, 1998; Davis et al., 2001; Komatsu et al., 2018). At high concentration (>1 µmol) NO is known to have a cytotoxic effect due to formation of the highly reactive compound peroxynitrite (Ferrer-Sueta et al., 2018). At low concentrations (<1 µmol), NO exhibits cytoprotective properties and maintains the homeostasis of the cardiovascular and nervous systems (Davis et al., 2001; Naseem, 2005; Komatsu et al., 2018; Raffetto et al., 2019; Tegeder, 2019). The cardioprotective functions of NO are aimed at regulating blood pressure, vascular tone, inhibit platelet aggregation and leukocyte adhesion, and prevent proliferation of smooth muscle cells (Xia and Vanhoutte, 2011; Omar et al., 2016; Hou et al., 2019; Taysi et al., 2019). Decreased production and bioavailability of NO is a hallmark of many underlying chronic diseases, including hypertension, ischemia–reperfusion injury, atherosclerosis, and diabetes (Ferrer-Sueta et al., 2018). Violation of the bioavailability of NO leads to a loss of cardioprotective effect and, in some cases, can even enhance the progression of the disease. This deficiency of NO is mainly caused by impaired functioning of NO synthetases and increased NO uptake due to the formation of reactive oxygen species (ROS).

Currently, in cardiology for the treatment of coronary heart disease, drugs that are donors of NO are widely used (Keefer, 2011; Hou et al., 2019; Xia and Vanhoutte, 2011). NO is a cardioprotective mediator in various cardiological processes, such as ischemia, hypertension, stroke, and others (Xia and Vanhoutte, 2011). Recently, a novel mitochondria-targeted superoxide-responsive donor NO was developed, providing significant protection against ischemic/reperfusion damage in rat cardiomyocytes (H9c2 cells) and isolated rat hearts (Hou et al., 2019).

The most common drugs in term of treatment cardiovascular disease are nitroglycerin, nitrosorbid, nitroprusside, and nitrite, which release NO in cells and tissues during their metabolism (Münzel and Schulz, 2010; Kapelko et al., 2017; Gatzke et al., 2018; Liu et al., 2019; Lu et al., 2019; Zhao et al., 2019). However, these drugs are often unstable, non-specific, toxic, and have a number of side effects (Tardiolo et al., 2019). Common known side effects of nitrate therapy include headache, flushing, lightheadedness, and postural hypotension (Divakaran and Loscalzo, 2017). In addition these drugs have many others disadvantages, for example, sodium nitroprusside demonstrated cyanide toxicity, altered mental status, seizure, metabolic acidosis, and nitrosorbide, nitroglycerin can induce methemoglobinemia, fatigue, and lethargy (Paul et al., 1986; Shumaev et al., 2018). In recent years, the interest in the study of nitrosyl complexes of transition metals, particularly iron complexes possessing cytoprotective properties, increases exponentially (Lewandowska et al., 2011). However, such compounds are often poorly soluble in water, short-lived and toxic to the body (Kapelko et al, 2017). Additionally, molecular mechanism of their action and the metabolic effects in cells are not well understood. Therefore, the development of new cardiac drugs with new improved properties is very important for the treatment of cardiovascular diseases. Additionally, cardiac failure often develops during the treatment of cancer patients because of the high toxicity of anticancer drugs (Reis-Mendes et al., 2015; Kuriakose et al., 2016; Hrynchak et al., 2017; Cuomo et al., 2019; Wenningmann et al., 2019). Heart failure is one of the most harmful clinical manifestations of cardiotoxicity, and heart failure can occur immediately or appear years after chemotherapy treatment.

Therefore, the development of new cardiac drugs with cardioprotective properties without side effects that can be used to treat cancer with the aim of maintaining the function of the cells of the cardiovascular system and the whole organism is of particular importance. These drugs can be used in cardiac oncology to reduce the cardiac toxic effects of chemotherapy drugs. A development of new strategies for reduction of cardiac toxicity has great clinical impact.

Thus, the synthesis of drugs that reduce the toxic effects of chemotherapy and enhance the viability of normal cells (fibroblasts and cardiomyocytes) is a relevant problem in cardiology and oncology.

The purpose of this study was to synthesize water soluble cationic dinitrosyl iron complexes (DNICs), to study their cytoprotective properties *in vitro* (in particular, the effect on the viability and metabolic processes in human lung fibroblasts and rat cardiomyocytes, and assess the efficiency of the therapeutic action of DNICs. The objectives of this study were to establish the effect of DNICs on mitochondrial membrane potential, ATP synthesis, glutathione level, ROS, viability and proliferation of human lung fibroblasts and rat cardiomyocytes.

## Materials and Methods

### Reagents

In this work, the following reagents were used: Dulbecco’s Modified Eagle Medium (DMEM, low glucose-1 g/l for culturing fibroblasts), L-glutamine, 25 mM HEPES, sodium pyruvate, fetal bovine serum (FBS, ultra-low endotoxin content), Trypsin 0.25%, EDTA 0.02% in HBSS, Gentamicin (10 mg/ml) were purchased from Biowest (France). DMEM (high glucose-4.5 g/l for culturing rat cardiomyocytes) was purchased from OOO NPP PanEko (Russia). AlamarBlue^®^ Cell Viability Reagent; 5,5′,6,6′- tetrachloro-1,1′,3,3′- tetraethylbenzinidazolylcarbocyanine iodide (JC-1 dye); 2′, 7′- dihydrodichlorofluorescein diacetate dye (H2-DCFDA); propidium iodide (PI) were purchased from Molecular Probes Company (Eugene, USA). O-phthalaldehyde was purchased from Fisher Scientific Company (Loughborough, UK).

### Cell Lines

The human lung embryonic fibroblasts (HLEF, cell line HLEF-104) were purchased from BioloT (St. Petersburg, Russia). Rat cardiomyocytes (cell line H9c2) were purchased from ATCC (Manassas, Virginia, Merck, USA).

### Cell Culture

Cells were cultured in DMEM supplemented with 10% fetal bovine serum (FBS, vol/vol), glutamine (0.15%), HEPES (10 mM, pH 7.2) and gentamicin (50 mg/ml).

Cells were grown in plastic tissue culture flasks (Corning Incorporated, Corning, NY, USA) in an atmosphere of 5% CO_2_ at 37 °C and 90% humidity. Cells were reseeded twice a week. For all experiments, cells from exponentially growing cultures were used. After the cells in the monolayer reached a density of 90%, they were treated with 0.25% trypsin and EDTA and centrifuged at 3,000 g for 5 min. The supernatant was discarded, and the cell pellet was re-suspended in growth medium and the cells were plated in a 96-well plate. All experiments were performed using an HLEF-104 culture <20 passages and an H9c2 culture <25 passages.

### Preparation of Cell Lysates

Human lung embryonic fibroblasts (cell line HLEF-104) were cultured in DMEM medium with 10% FBS at 37 ºC and 5% CO_2_. Cells were grown to a density of 90% in culture flasks. When confluence of 90% was achieved, cells were treated with 0.25% trypsin-EDTA, precipitated by centrifugation at 3,000 g for 5 min. Then, the resulting cell pellet was re-suspended in phosphate buffer (PBS, 0.1 M, pH 7.4), and cell lysates were prepared. The cell lysates were prepared by five times pushing the cells PBS (0.1 M, pH 7.4.) through a needle of a syringe (needle diameter was 25 G). The protein concentration (1.5 mg/ml) was determined with the Biuret test. The cell lysates were used to determine the activity of ATPase and total amount of ATP.

### Synthesis of the DNICs

Water-soluble cationic mononuclear DNICs (#3-[Fe(SC(NH2)2)2(NO)2]2Fe2(S2O3)2NO4; #4 -[Fe(SC(NH2)(NHC2 H5))2 (NO2)]Cl[Fe(SC(NH2)(NHC2H5 ))Cl(NO2)]; #6-[Fe(SC(NH2)2)2(NO)2]ClO4Cl) with functional sulfur-containing ligands, thiourea were synthesized as described in protocol (Sanina et al., 2015; Sanina et al., 2019). The structure of DNICs was studied by X-ray analysis, Mössbauer, IR and EPR spectroscopy (Emel’yanova et al., 2015; Sanina et al., 2015; Shmatko et al., 2017b). When dissolved in water solvents, these DNICs release NO as a result of dissociation (Sanina et al., 2015).

### Electrochemical Determination of NO, Generated From DNICs

The amount of NO generated by DNICs (#3, #4 and #6) in solution was carried out using sensor electrode “amiNO-700” of the system “in NO Nitric Oxide Measuring System” (Innovative Insruments, Inc., Tampa, FL, USA) in accordance with the previously described method (Sanina et al., 2015).

### Cell Viability Assay

The effect of DNICs on the cell viability was analyzed using the AlamarBlue^®^ Cell Viability Assay (ThermoFisher Scientific, United States). This method makes it possible to determine the activity of mitochondrial NADH dehydrogenases, which cleave NADH to NAD and H^+^, and the formed proton reduces the dye resazurin to fluorescent rezofurin (Schreer et al., 2005). Fibroblasts (4,000 cells per well) or rat cardiomyocytes (4,500 cells per well) were seeded on a 96-well plate and grown overnight in an incubator atmosphere of 5% CO_2_ at 37 °C and 90% humidity. The next day, the growth medium was replaced with a new one in the wells and DNICs (2 × 10^-4^ M) were added to cells. DNICs (2 × 10^-4^ M) were dissolved in water immediately prior to adding, and an equal volume of PBS (0.1 M, pH 7.4) was added to the control wells. Then plate with samples was incubated at 37 °C for 20 min. Thereafter, doxorubicin (1.4 × 10^-4^ M) was added to the cells. The plate with samples were again incubated at 37 °C for 20 min. AlamarBlue^®^ reagent (10 µl) was then added to the cells in each well, and fluorescence intensity was measured for 35 h at E_ex_/E_em_ = 570/590 nm using a Varian Cary Eclipse spectrofluorimeter (Agilent Technologies, United States). Data are presented as the average of three repeated experiments.

### Determination of Mitochondrial Membrane Potential

DNIC-induced mitochondrial membrane potential changes (ΔΨ_m_) were determined using a fluorescent lipophilic cationic probe 5, 5′,6, 6′-tetrachloro-1,1′,3,3′- tetraethylbenzinidazolylcarbocyanine iodide, JC-1dye. This method is based on the fact that JC-1 accumulates in mitochondria in proportion to ΔΨ_m_, forming aggregates that exhibit red fluorescence. However, in the cytoplasm, JC-1 exists as monomers, and exhibits green fluorescence. It has been established that the ratio of red to green fluorescence is proportional to ΔΨ_m_ (Lisa et al., 1995; Ganguly et al., 2010). Fibroblasts (4,000 cells per well) or rat cardiomyocytes (4,500 cells per well) were supplemented with a solution of DNICs (2.0 × 10^-4^ M), the control wells were supplemented with an equal volume of PBS (0.1 M, pH 7.4.), and the plates were incubated for 15 min. Then, JC-1 (2.6 × 10^-5^ M) was added in the dark, and the plates were incubated at 37 °C for 30 min. Intensity of red fluorescence (excitation, 570 nm; emission, 595 nm) and green fluorescence (excitation, 485 nm; emission, 535 nm) was measured using a Varian Cary Eclipse spectrofluorimeter (Agilent Technologies, USA) (Ganguly et al., 2010). Data are presented as the average of three repeated experiments.

### Intra Cellular ROS Accumulation Study

The level of ROS formation in cells by the action of DNICs was assessed by fluorometric method, using 2,7′-dihydrodichlorofluorescin diacetate (H2-DCFDA, Molecular Probes, USA) (Oksvold et al., 2002). H2-DCFDA is a nonfluorescent, cell-permeant compound. When H2-DCFDA enters the cell, endogenous esterases within the cell cleave the acetate groups, thereby capturing the reduced form of the probe (DCHF) intracellularly. It is known that the probe can be readily oxidized to DCF by H_2_O_2_ or OH^-^ to form a fluorescent compound. Briefly, DNICs (2.0 × 10^-4^ M) were added to fibroblasts (4,000 cells per well) and rat cardiomyocytes (4,500 cells per well), and equal volumes of PBS (0.1 M, pH 7.4) were added to the control, then the plate was incubated for 15 min at 37 °C. The cells were then washed twice with PBS (0.1 M, pH 7.4), then H2-DCFDA (10 µmol, 10 µl) was added to the wells and incubated for 30 min at 37 °C in the dark. Then the cells were washed twice in PBS (0.1 M, pH 7.4) at 24 °C for 5 min. After that, the fluorescence intensity was measured at wavelengths of excitation and emission of the oxidized form, at 488 nm and 525 nm, respectively.

Data are presented as the average of three repeated experiments.

### Determination of Intracellular GSH Contents

The influence of DNICs on the level of cellular GSH was carried out by modification of a previously published method (Hissin and Hilf, 1976; Banerjee et al., 2017). The effect of DNICs on the level of intracellular reduced glutathione was assessed using o-phthalaldehyde. The principle of the method is that the amino groups and sulfhydryl groups of glutathione react with o-phthalaldehyde and reduce it to form a fluorescent product. Briefly, fibroblasts (4,000 cells per well) and rat cardiomyocytes (4,500 cells per well) were seeded into a 96-well plate and cells were grown for 24 h in an incubator at 37 °C, 5% CO_2_. Then, cells were treated with DNICs (2 × 10^-4^ M), the control samples were supplemented with an equal volume of PBS (0.1 M, pH 7.4.) and the plates were incubated for 15 min at an incubator at 37 °C, 5% CO_2_. Then the cells were washed twice with PBS (0.1 M, pH 7.4) and a solution of cold distilled water (400 µl) containing 17.5% HPO_3_ was added to them. After incubation for 10 min the solution was removed and o-phthalaldehyde (100 µl, 0.1% in ethanol) was added to the cells, incubated for 15 min at 37 °C. After that, the fluorescence adduct GSH was determined at wavelengths (excitation, 350 nm; emission, 420 nm) using a Varian Cary Eclipse spectrofluorimeter (Agilent Technologies, USA) (Ganguly et al., 2010). Data are presented as the average of three repeated experiments.

### Cytotoxicity of DNICs

Cytotoxicity of DNICs was determined by staining the cells with the aniline dye trypan blue (Venkataraman, 1956). Human lung embryonic fibroblasts (cell line HLEF-104) were grown in culture flasks in DMEM growth medium in an incubator at 37 °C in a 5% CO_2_ atmosphere. The number of living and dead cells was counted by staining the cells with trypan blue. To assess the cytotoxicity of DNICs, cells (25,600 cells/ml) were supplemented with DNICs (2 × 10^-4^ M) and incubated for 24 h. HLEF cells (25,600 cells/ml) without DNICs served as a control. Then cells were treated with 0.25% trypsin-EDTA, aliquots (0.2 ml of the cell suspension) were mixed with a trypan blue solution (0.1 ml of 0.5% solution in 0.9% NaCl) and incubated with the dye for 10 min at 37 °C. The number of living and dead cells and the total number of cells was determined in a standard hemocytometer chamber. The cytotoxic index (CTI) was calculated by the formula: (number of dead cells/total number of cells) × 100%. Data are presented as the average of three repeated experiments.

### Cell Cycle Analysis

The effect of DNICs on the cell cycle was studied by flow cytometry (Ormerod et al., 1998). Briefly, fibroblasts were grown in culture flasks to a density of 10^6^ cells. Then DNICs (2 × 10^-4^ M) were added to the experimental vial, and an equal amount of growth medium was added to the control vial (positive control) and incubated for 24 h at 37 °C in 5% CO_2_. After this, the cells were pelleted by centrifugation at 3,000 g for 5 min, the supernatant was discarded, and the cell pellet was washed with PBS (0.1 M, pH 7.4). Then the cells were fixed cooled to -20 °C with 70% ethanol (2 ml) and incubated for 4 h at -20 °C. After incubation, the cells were washed twice in PBS (0.1 M, pH 7.4.), incubated for 30 min at 24 °C with RNase A (0.1 mg/ml) and propidium iodide (0.01 mg/ml), Triton X-100 (0.1%). A portion of the cells was left as negative control (unstained cells). The cell cycle distribution was determined using a FACS (Calibur Flow Cytometer, Becton Dickinson, USA). After staining, samples were analyzed using flow cytometry on a Guava EasyCyte System (Millipore) Guava^®^ Cell Cycle. The fluorescence intensity of propidium iodide was measured at E_x_/E_m_ = 488/585 nm. The analysis was performed using Guava Soft ^™^ 3.1.1 (Millipore, USA). The fluorescence intensity of propidium iodide was presented on the histogram in the G0/G1, S and G2/M phases of the cell cycle. Ten thousand events were analyzed for each sample. Appropriate gating was used to select the single-cell population. The same gate was used on all samples, ensuring that the measurements were taken on a standard cell population. Data are presented as the average of three repeated experiments.

### Measurement of ATPase Activity

ATPase activity was measured in cell lysates by the colorimetric method using the ATPase activity analysis kit (BioVision Kit, USA) in accordance with the attached manual. Briefly, whole cells (2 × 10^6^) were quickly homogenized with a buffer (400 µl) cooled on ice and the samples were placed on ice for 10 min. Then the samples were centrifuged at 10,000 g at 4 °C for 10 min and the supernatant was collected. Endogenous phosphate removed by using ammonium sulfate method. Then samples (20 µl, protein 1.5 mg/ml) added in triplicates onto a clear 96-well plate (labeled “background control”, and “sample ATPase activity”). DNICs (2 × 10^-4^ M) were added to tested samples and incubated 15 min on ice. For reagent control ATPase assay buffer (100 µl) added. ATPase Positive Control (10 µl) diluted into of ATPase assay buffer (190 µl). Then of ATPase Positive Control (20 µl) added into wells and adjusted final volume to 100 µl with ATPase assay buffer. For each well, 100 µl of a reaction mixture containing ATPase assay buffer (98 µl) and ATPase substrate (2 µl) was prepared. Then 100 µl of the reaction mixture was added to each well, for a positive control, reagent control and test samples, and incubated at 25 °C for 30 min. Measurement: added 30 µl ATPase assay developer to all standards, ATPase positive control and test samples and sample background controls, incubated at 25 °C for 30 min and measured absorbance (OD) at 650 nm at the end of incubation time. Calculation: plotted the phosphate standard curve. The sample background is corrected by subtracting a higher value obtained from background control or reagent control from all sample readings. The ATPase activity of the tested samples was calculated: ΔOD = A2 - A1. We used ΔOD to the standard phosphate curve to obtain B nmol of phosphate generated by ATPase during the reaction time (e.g. t = 30 min). The formula was used to calculate the activity of an ATPase sample = B/(tX V) × D = nmol/min/µl = mU/µl = U/ml. Where: B is the amount of phosphate from the standard curve (nmol), t is the reaction time (min), V is the volume of the sample added to the reaction well (µl), D is the dilution factor of the sample. Unit definition: one ATPase unit is the amount of enzyme that will generate 1.0 µmol phosphate per min at pH 7.5 at 25 °C. Data are presented as the average of three repeated experiments.

### Measurement of Total ATP

The total amount of ATP was measured in cell lysates by a fluorometric method using the ab83355 ATP assay kit (Abcam, USA) in accordance with the manual. The analysis is based on phosphorylation of glycerol in order to obtain a product that can be easily quantified fluorometrically (E_x_/E_m_ = 535/587 nm) (Gregorczyk et al., 2018). Briefly, fibroblasts were seeded and grown in culture flasks to a density of 10^6^ cells and treated with DNICs or untreated (positive control). DNICs (2 x 10^–4^ M) was added to the experimental flask, and an equal amount of growth medium was added to the control flask and incubated for 15 min at 37 °C and in 5% CO_2_. Then prepared cell lysates according to protocol. The growth medium was removed and the cells (1 × 10^6^) were washed with cold PBS (0.1 M, pH = 7.2) and re-suspended in 100 µl of ATP assay buffer. Then the cells were destroyed by pipetting up and down a few times. After this, the cells were centrifuged for 5 min at 4 °C at 13,000 g in a cold micro-centrifuge to remove any insoluble material. Supernatants collected and transferred to new tubes, kept on ice. Cells samples may contain enzymes that can interfere with the assay. To remove these enzymes from samples Deproteinizing Sample Preparation Kit – TCA (ab204708, USA) has been used. Then the samples (50 µl, protein 1.5 mg/ml) were added to a 96-well plate (labeled “background control” and “ATP samples”), and adjusted final volume to 100 µl with ATP assay buffer. For each well, 50 µl of the reaction mixture containing ATP assay buffer (45.8 µl) + ATP probe (0.2 µl), ATP converter (2 µl), developer mix (2 µl) was prepared and added to each well. The background reaction mixture was the same, but without the ATP converter (in the absence of an ATP converter, the analysis detects only glycerol phosphate, but not ATP).Then we added 50 µL of background reaction mix into the background control sample wells. Samples were mixed and incubated at room temperature for 30 min in the dark. Then immediately measured output on a microplate reader at E_x_/E_m_ = 535/587 nm. Calculations: subtracted the sample background control from sample readings. The adjusted values for each standard are plotted against the final ATP concentration. Standard curve was used for adjusted relative fluorescence of the sample to obtain the amount of ATP (B) in the sample wells. Concentration of ATP (nmol/µl or µmol/ml or mM) in the test samples was calculated as: ATP concentration = (V/B × D) × DDF. Where: B = amount of ATP in the sample well calculated from standard curve (nmol or mM); V = sample volume added in the sample wells (µl); D = sample dilution factor if sample is diluted to fit within the standard curve range (prior to reaction well set up); DDF = deproteinization dilution factor. Data are presented as the average of three repeated experiments.

### Statistical Analysis

The analyses were repeated at least three times. Mean ± standard deviation (SD) was adopted to present the results. For comparison between control and treatment groups, data were calculated using one-way analysis of variance (ANOVA) followed by the Tukey’s test. A value of P ≤ 0.05 was considered significant. All experiments were repeated three times.

## Results

### Synthesis of DNICs

**Figure 1:**
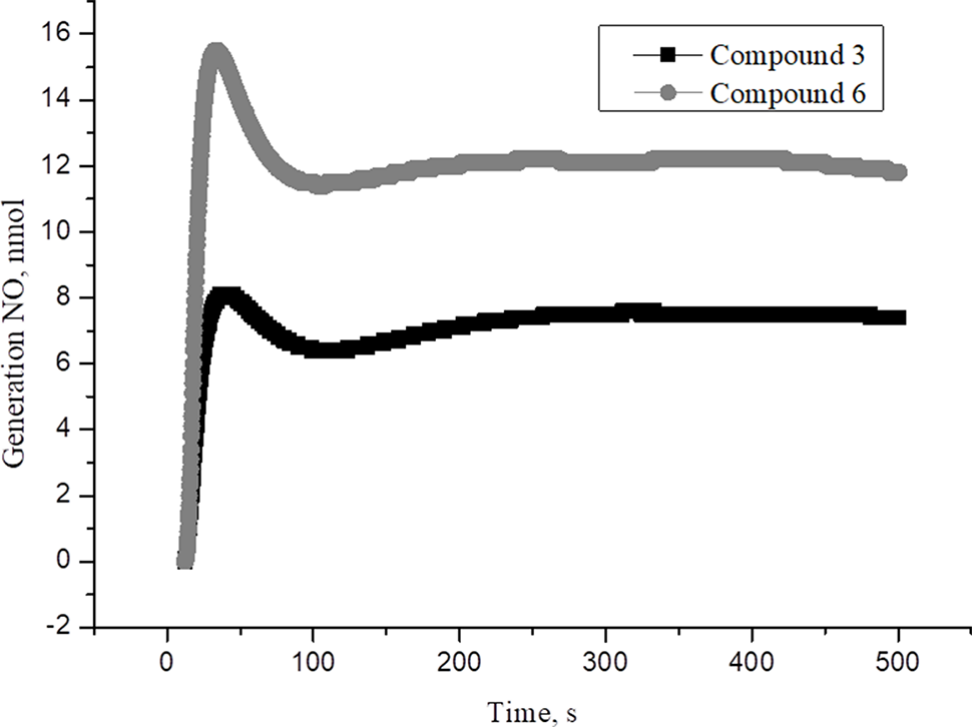
The time dependence of NO amount generated by DNIC#3 and DNIC#6 in aqueous solutions at pH 7 and T = 25 °C under anaerobic conditions. The DNICs concentration was (2 × 10^-4^ M). The values presented are the means ± SD of three independent experiments, n = 3.

**Figure 2:**
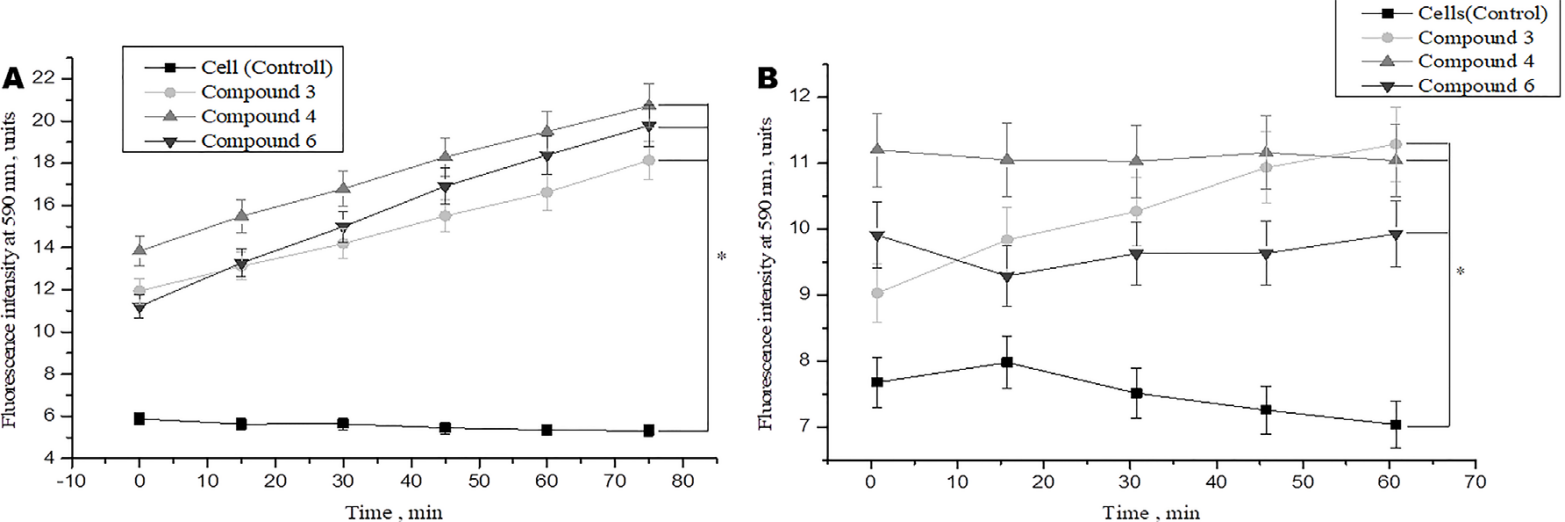
Effect of DNICs on the cell viability of human lung embryonic fibroblasts (cell line HLEF-104) **(A)** and rat cardiomyocytes (cell line H9c2) **(B)** for 60 min. DNICs (2 × 10^-4^ M) were added to cells, then cell viability was tested in fibroblasts and rat cardiomyocytes by AlamarBlue^®^ Cell Viability Assay. *P ≤ 0.05 vs. control group, with ANOVAs followed by Tukey’s *post hoc* test. The values presented are the means ± SD of four independent experiments, n = 4.

**Figure 3:**
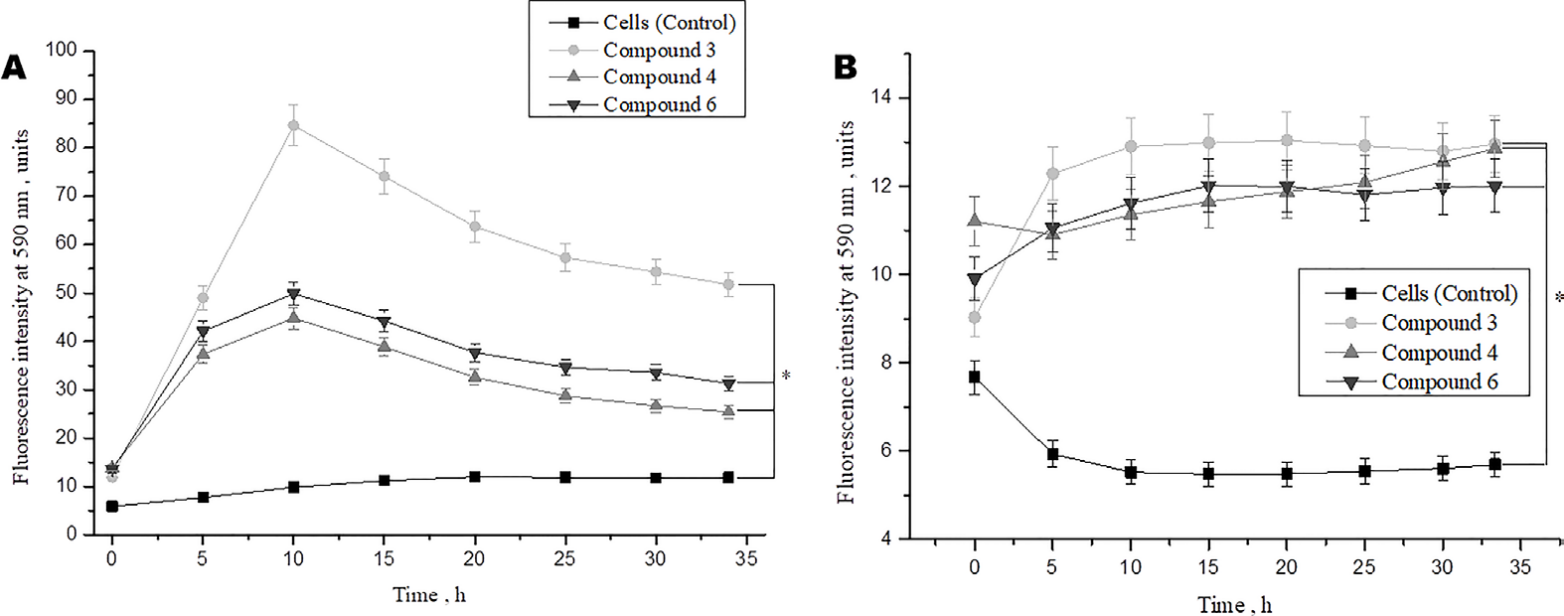
Effect of DNICs on the cell viability of human lung embryonic fibroblasts (cell line HLEF-104) **(A)** and rat cardiomyocytes (cell line H9c2) **(B)** for 35 h. DNICs (2 × 10^-4^ M) were added to cells, then cell viability was tested in fibroblasts and rat cardiomyocytes by AlamarBlue^®^ Cell Viability Assay. *P ≤ 0.05 vs. control group. The values presented are the means ± SD of four independent experiments, n = 4.

**Figure 4:**
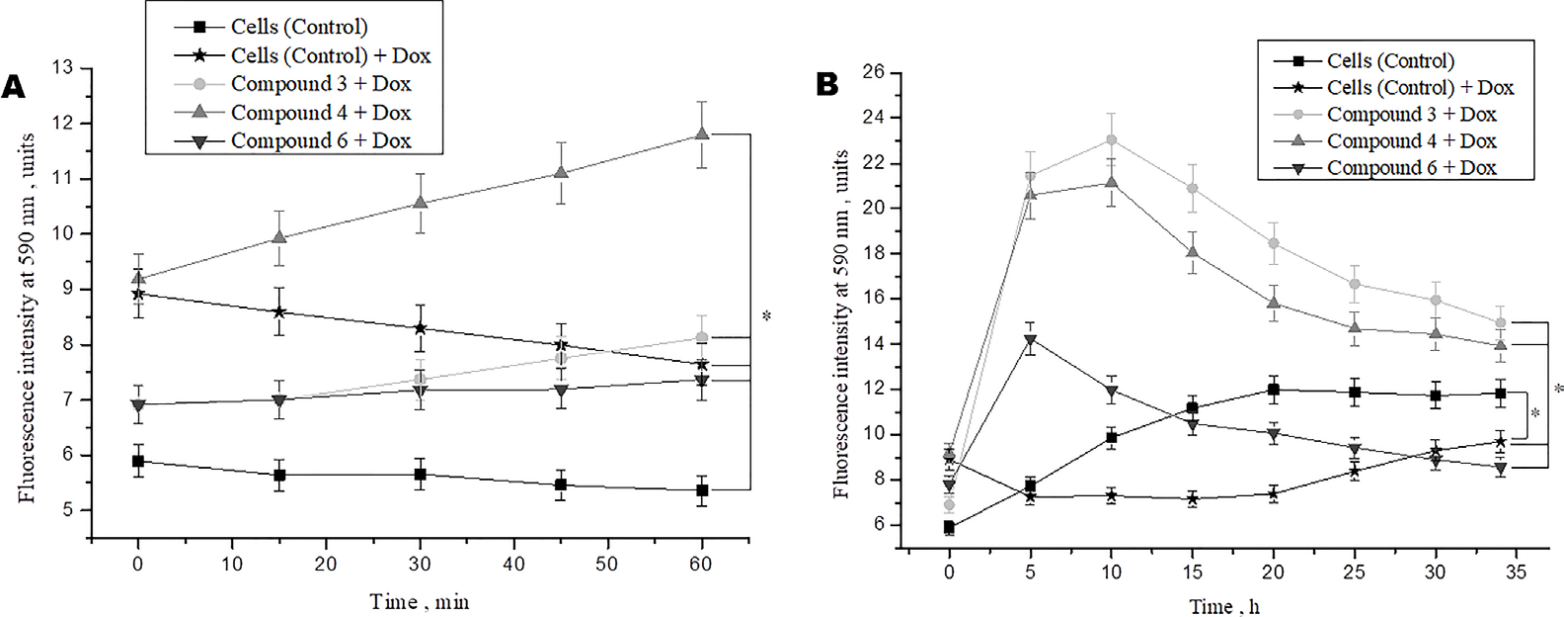
Effects of DNICs on cell viability of fibroblasts, treated with doxorubicin for 60 min **(A)** and 35 h **(B)**. DNICs (2 × 10^-4^ M) were added to HLEF-104 cells, then doxorubicin (1.4 × 10^-4^ M, 20 min after DNICs treatment) was added, cell viability was tested in fibroblasts by AlamarBlue^®^ Cell Viability Assay after doxorubicin-induced toxicity. *P ≤ 0.05 vs. control group. The values presented are the means ± SD of four independent experiments, n = 4.

New family of cationic water soluble DNICs with S-donor ligands, such as thiocarbamide and its derivatives was obtained and characterized (Sanina et al.,2014, Sanina et al., 2015; Sanina et al., 2016; Shmatko et al., 2017a). These complexes are models of the active sites of nitrosyl non-heme [Fe–S] proteins existing in all living organisms, from bacteria to mammals (Butler and Megson, 2002; Lewandowska et al., 2011). Their structure and properties have been studied by X-ray analysis, IR-, Mössbauer-, and EPR-spectroscopy (**Figure 1, Supplementary Material**) (Shmatko et al., 2017b). It was shown that these DNICs are effective NO donors and have more than twice high NO-generating ability compared to the commercial NO-donor diethylenetriamine (**Figures 2**–**4, Supplementary Material**) (Sanina et al., 2014; Sanina et al., 2015). In this study, DNICs were synthesized according to the previously described protocol (Sanina et al., 2015). Then, NO release from DNICs complexes was investigated. Weighted portions (2×10^-4^ M) DNICs were prepared and dissolved in water. After that, the formation of NO was measured using the electrochemical method. First, the release of nitric oxide was analyzed using the DNIC # 3 complex (**Figure 1**). As can be seen from **Figure 1**, when DNIC # 3 was dissolved in water, 8 nmol of NO was released for 50 s, then after 100 s the level of NO dropped to 7 nmol and remained constant for 500 s. The release of NO from DNIC#6 in an aqueous solution was also investigated (**Figure 1**). It was shown that when DNIC#6 was dissolved in an aqueous solution, 16 nmol NO was released for 50 s, then after 100 s the NO level dropped to 12 nmol and remained constant for 500 s. These results showed that when dissolving DNICs in water, the actual concentration of released NO is 7–12 nmol for 500 s.

### Effect of DNICs on Cell Viability of Fibroblasts and Rat Cardiomyocytes

Studies have shown that various types of NO donors spontaneously generate NO under physiological conditions and exhibit protective effects against ischemia or reperfusion injury in cardiomyocytes (H9c2 cell line) and isolated rat hearts (Hou et al., 2019; Webb et al., 2004). We studied the short-term and long-term effects of DNICs on cell viability. In this study, two cell lines (fibroblasts and rat cardiomyocytes) were used to determine whether DNICs possess cytotropic properties. The effect of DNICs (#3, #4, and #6) on cell viability was evaluated using the AlamarBlue^®^ Cell Viability fluorescence assay. As can be seen from **Figure 2A**, DNICs (#3, #4 and #6) increased the viability of fibroblasts, since after 15 min there was an increase in fluorescence intensity. The viability of rat cardiomyocytes also increased 15 min after treatment with DNICs, compared to control cells not treated with these compounds (**Figure 2B**).

We also investigated the effect of DNICs on cell viability over time (up to 35 h). The results showed that the viability of fibroblasts under the action of DNICs (#3, #4, and #6) gradually increased and reached its maximum value after 10 h (P < 0.05, **Figure 3A**). Compound # 3 increased the viability of fibroblasts significantly (∼ 8 times), and compounds #4 and #6 increased it 4.5 times, compared to the control cells that were not treated with DNICs. Then after 10 h viability slowly decreased, however, remained higher than in cells untreated with DNICs (**Figure 3A**). It was found that DNICs (#3, #4, and #6) also increased the viability of rat cardiomyocytes (P < 0.05, **Figure 3B**). As can be seen from **Figure 3B** the viability of rat cardiomyocytes after treatment with DNICs increased by 2 fold, compared with control cells without treatment with these compounds. After 10 h, the viability of rat cardiomyocytes treated with DNICs reached a maximum value (12 relative units). Thus, long incubation of rat cardiomyocytes with DNICs led to a significant increase in their viability and even after 35 h it remained at a high level compared to control cells (**Figure 3B**). The exact values of the fluorescence intensity after short-term and long-term treatment of DNICs cells are presented in **Table 1**. These results demonstrated that DNICs increased significantly the cell viability of fibroblasts and rat cardiomyocytes, suggesting the DNICs have cytotropic properties. Since the AlamarBlue^®^ Cell Viability Assay was based on the determination of the activity of mitochondrial dehydrogenases, we have shown that DNICs increased the activity of mitochondrial NADH dehydrogenases, which play a central role in cellular energy production (Efremov et al., 2010). Therefore, these results indicate that DNICs are cytotropic agents.

**Table 1:** The effect of DNICs on cell viability over time (15 min, 60 min, and 10 h).

Name of experiment	Fibroblasts			Fibroblasts with doxorubicin			Cardiomyocytes		
Time	15 min	60 min	10 h	15 min	60 min	10 h	15 min	60 min	10 h
Cell (Control)	5,639	5,355	9,869	8,592	7,642	7,311	7,979	7,035	5,527
DNIC#3	13,135	16,622	84,602	7,004	8,122	23,033	9,837	11,290	12,90
DNIC#4	15,498	19,497	44,838	9,927	11,795	21,148	11,053	11,043	11,361
DNIC#6	13,290	18,401	45,315	8,357	10,187	11,982	9,290	9,930	11,618
Negative control	1, 209	1,213	1,230	1,215	1,250	1,244	1,210	1,225	1,216

Data are presented as mean ± SD of three independent experiments.

### Effects of DNICs on Doxorubicin-Induced Toxicity in Fibroblasts

Studies demonstrated that doxorubicin plays an important role in the cancer therapy-induced cardiomyopathy (Adão et al., 2013; Truong et al., 2014). To study the protective effects of DNICs on doxorubicin-induced toxicity, a doxorubicin-induced toxic model on fibroblasts was used. Cells were pretreated with DNICs and then supplemented them with doxorubicin, a well-known cytostatic anti-cancer agent, which is used to treat breast cancer (Agudelo et al., 2016). The results showed that short-term treatment of cells with doxorubicin caused a decrease in cell viability (**Figure 4A**). At the same time, incubation of cells with DNICs + doxorubicin showed an increase in cell viability over time (**Figure 4A**; 15, 60 min).This indicated that DNICs reduced the toxic effect of doxorubicin on cells, and this protective effect developed over time. It should be noted that the maximum toxic effect of doxorubicin was observed only after 10 h (P < 0.05, **Figure 4B**). Long-term treatment of cells with DNICs + doxorubicin increased cell viability and protected cells from the toxic effects of doxorubicin (P < 0.05, **Figure 4B**). The maximum effect of DNICs was also observed after 10 h. After 10 h, the cell viability was slowly reduced, however, even after 35 h the cells treated with DNICs had a higher viability compared to control cells (not treated with DNICs). All DNICs showed a protective effect however, DNICs #3 and #4 had the most pronounced protective effect. The exact values of the fluorescence intensity after treatment of DNICs+doxorubicin are presented in **Table 1**.

Thus, the results showed that doxorubicin caused a decrease in cell viability, whereas cells treated with DNICs maintained a higher viability, in particular, their survival was 2–3 times higher than that of untreated cells. These data indicate that with DNICs have cytoprotective properties. These results also showed that DNICs # 3, # 4 are the most effective protectors against doxorubicin-induced cytotoxicity in fibroblasts.

Therefore these data indicate that DNICs are effective long-acting cytoprotectors that protect cells from the toxic effects of doxorubicin.

### Effects of DNICs on the Mitochondrial Membrane Potential of Fibroblasts and Rat Cardiomyocytes

Mitochondrial dysfunction is reported to have occurred with ischemia (Galluzzi et al., 2009; Yang J-L et al., 2018; Liu et al., 2019). Reports show that some chemical compounds that can selectively accumulate in mitochondria, reducing their membrane potential, have protective properties and can be used to prevent severe disorders after ischemia and a stroke (Wang et al., 2009; Hou et al., 2019; Hu et al., 2019; Liu et al., 2019).

**Figure 5:**
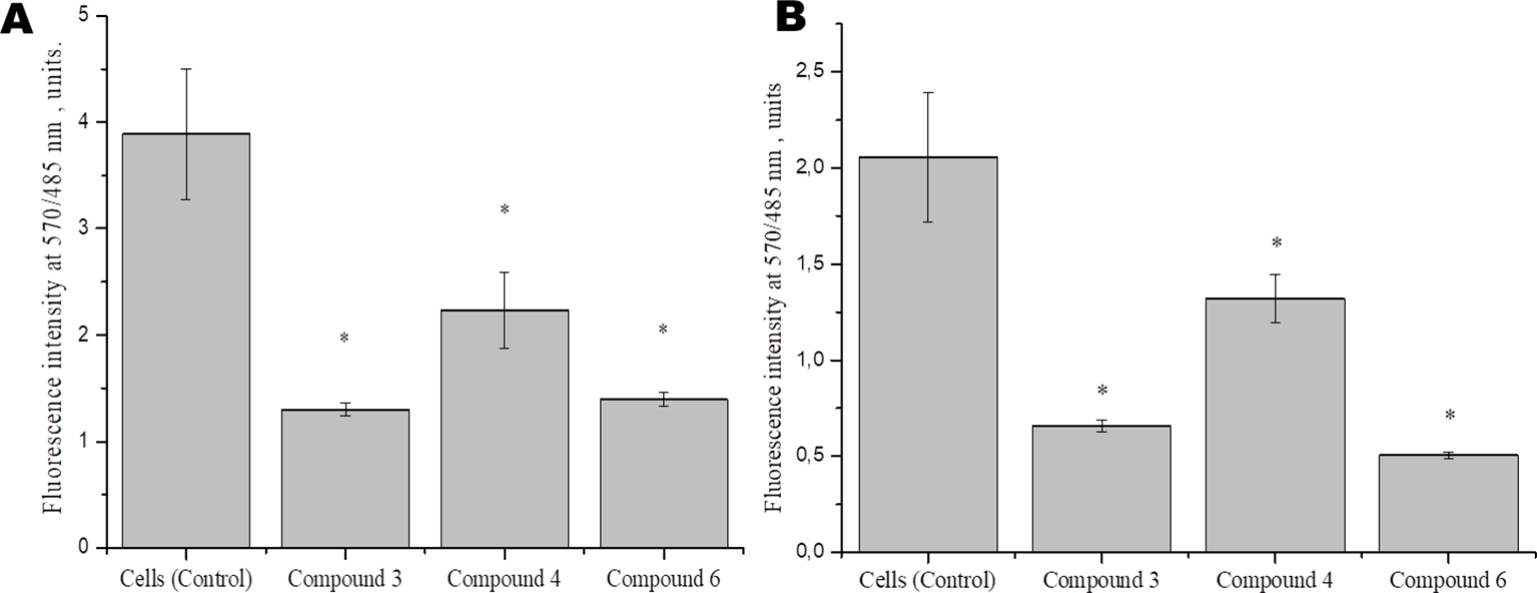
Effects of DNICs on the mitochondrial membrane potential of fibroblasts (cell line HLEF-104) **(A)** and rat cardiomyocytes (cell line H9c2) **(B)**. Cells were treated with DNICs (2 × 10^-4^ M) or without (control) for 15 min, then JC-1 was added. The ratio of red fluorescence (mitochondrial JC-1) to green fluorescence (cytoplasmic JC-1) was used to calculate the mitochondrial potential. *P ≤ 0.05 vs. control group. The values presented are the means ± SD of four independent experiments, n = 4.

To identify the effect of DNICs on mitochondrial function, we examined the effect of DNICs on mitochondrial membrane potential fibroblasts and rat cardiomyocytes. The results indicated that DNICs (#3, #4, and #6) decreased the ΔΨ_m_ in 2–3 times in fibroblasts (**Figure 5A**). Indeed, the results showed that DNICs (#3, #4 and #6) also reduced the level of ΔΨ_m_ by 2–3 times in rat cardiomyocytes (**Figure 5B**). Above all, these results showed that DNICs affected on the mitochondrial function of cells. We suppose that DNICs are organizing an excess charge leakage through the mitochondrial membrane.

### Effect DNICs at Cell Cycle of Fibroblasts

**Figure 6:**
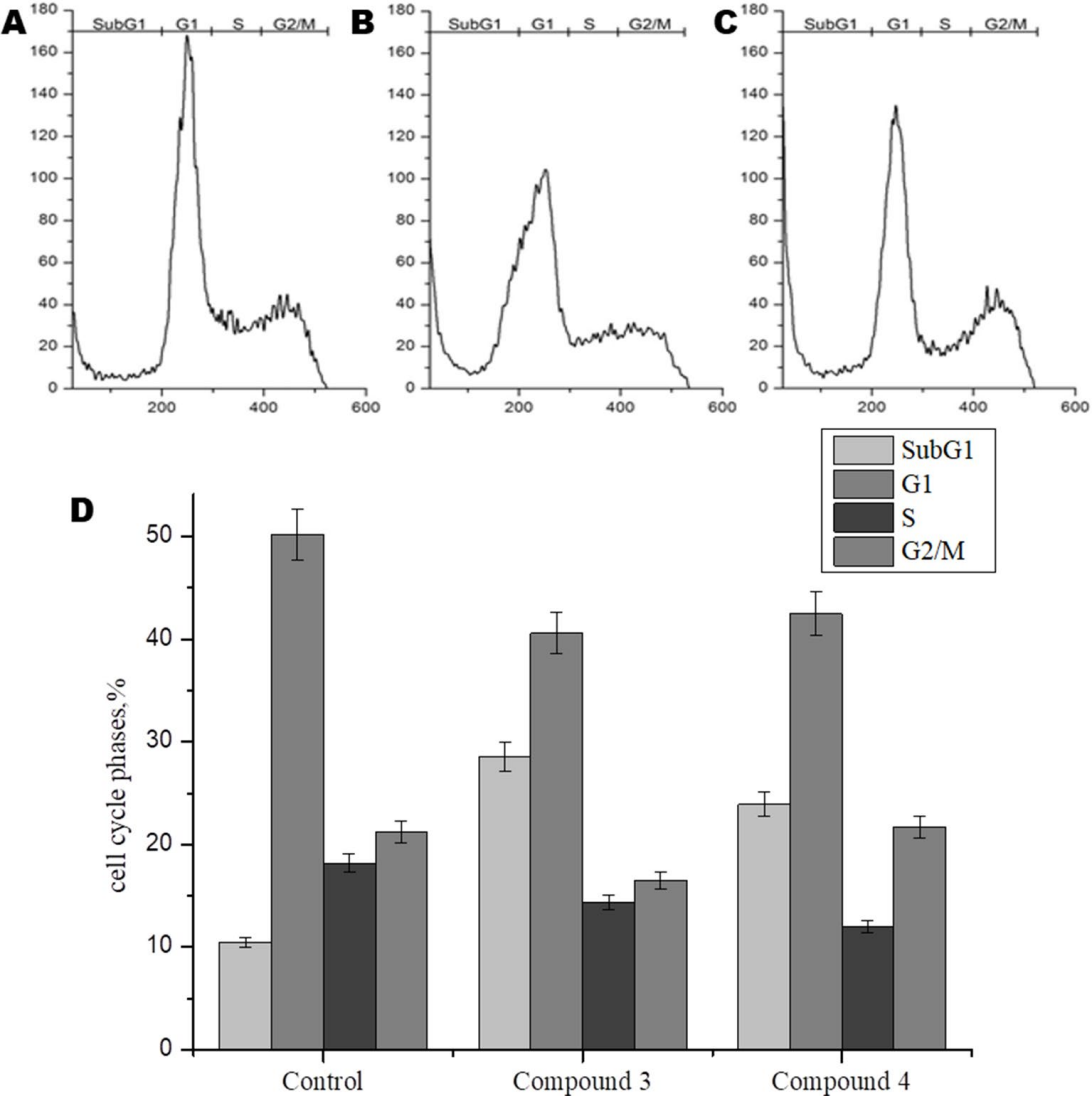
Flow cytometric analysis of effect of DNICs on cell-cycle of HLEF-104 cells. The cells were treated with DNICs (2 × 10^-4^ M) and untreated (control) for 24 h, samples were analyzed using FACS (Calibur Flow Cytometer, Becton Dickinson, USA). The cell cycle phase profile of HLEF-104: **(A)** Control, **(B)** DNIC#3, **(C)** DNIC#4. **(D)** Graphical presentation of distribution (%) of cells in different phases of cell cycle. The values in the bars indicates the percent population of cells in respective cell cycle phases. Results are presented as mean ± SD of three independent experiments.

In order to further study the mechanism of action of DNICs, we investigated the effect of these compounds on the cell cycle of fibroblasts by flow cytometry. Cells were seeded in culture flasks, and before sowing, living and dead cells were counted by trypan blue staining (Ruano et al., 2003). The results showed that before DNICs treatment 93.75% of the cells were alive and only 6, 25% were dead (**Table 2**). Then DNICs #3, #4 were added to the experimental flasks, and growth medium was added to the control flasks. After incubation for 24 h, the cells were analyzed by flow cytometry. In parallel aliquots of cell suspension were taken before cell fixation and the number of living and dead cells was counted again using trypan blue staining. The results showed that the number of dead cells after treatment with DNIC#3 was 7.8%, and after processing with DNIC#4 was 9.7% (**Table 2**). These data indicate that DNICs are not cytotoxic compounds. Then, the effect of these compounds on the fibroblast’s cell cycle was studied by flow cytometry. Using the histogram of fluorescence intensity, we isolated cells in the SubG1, G1, S, and G2/M phases of the cell cycle. As can be seen from **Figure 6**, the G1 and S phases were practically the same for the control and experimental samples (after incubating the cells with DNIC#3 and #4). Phase S, at which DNA replication of the cell nucleus occurs was 18.17% for the control sample, 14.35% for DNIC#3 and 11.95% for DNIC#4. However, these differences were not statistically significant (**Figure 6**). In addition, the phase of cell division (G2/M) for control cells was 21.21%, and for experimental cells after incubation with DNIC#4, this phase was 21.67%, and for DNIC#3 it decreased slightly to 16. 50% (**Figure 6**). However, no significant difference in the cell division (G2/M) was observed between control and experimental samples (P > 0.05).

**Table 2:** Determination of cytotoxicity of DNIC on fibroblasts.

Name of experiment	Total number of cells	Number of living cells	Number of dead cells/ml	Cytotoxicity index, %
Before the addition of DNIC#3 to cells	25,600	24,000	1,600	6.25
After the addition of DNIC#3 to cells	25,600	23,600	2,000	7.8
Before addition of DNIC#4 to cells	25,600	24,100	1,500	5.8
After addition of DNIC#4 to cells	25,600	23,100	2,500	9.7

Data are presented as mean ± SD of three independent experiments.

Thus, these data indicated that DNIC#3 and #4 were not affected the proliferation of fibroblasts and not stimulate their growth.

### Effect of DNICs on the Level of Glutathione in Fibroblasts and Rat Cardiomyocytes

**Figure 7:**
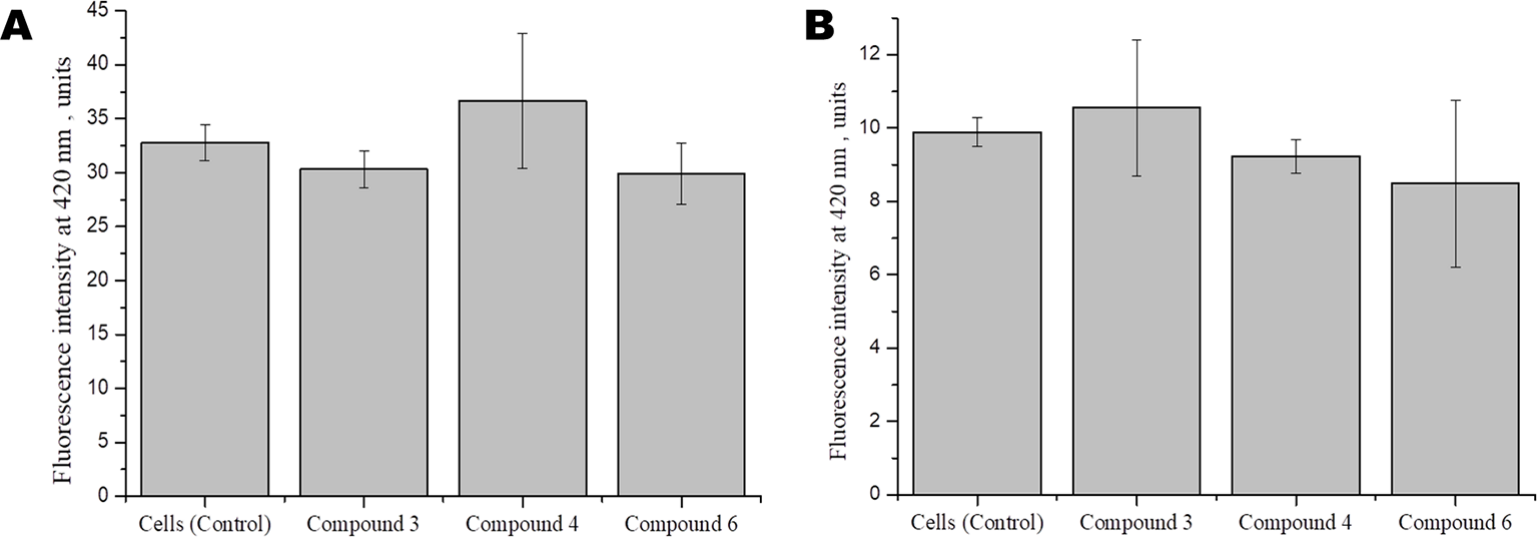
Effect of DNICS on the level of glutathione in fibroblasts **(A)**, in rat cardiomyocytes **(B)**. HLEF-104 or H9c2 cells were either kept untreated or treated with DNICs (2 × 10^-4^ M) for 15 min and intra cellular GSH was measured as described under *Materials and Methods* section. Results are presented as mean ± SD of three independent experiments. Differences between untreated control and DNICs treated cells are significant at *p ≤ 0.05 with ANOVAs followed by Tukey’s *post hoc* test.

Studies show that some cardiovascular drugs may also have antioxidant properties, as has been shown for some calcium channel blockers and β-adrenoceptor antagonists (Taddei et al., 2001). Several synthetic compounds, including free radical scavengers and free radical degrading agents, are currently under investigation in different clinical settings, including diabetes mellitus and acute ischemic stroke, in which neuroprotective effects have demonstrated (Margaill et al., 2005). To investigate whether DNICs have antioxidant properties, we assessed the effect of DNICs on the level of the reduced glutathione in fibroblasts and cardiomyocytes. The results showed that DNIC #3, #4, and #6 did not affect the level of the reduced glutathione either in fibroblasts or in cardiomyocytes (**Figures 7A, B**, respectively). Thus, DNICs maintained the intracellular GSH level.

### Effect of DNICs on the Level of Reactive Oxygen Species

**Figure 8:**
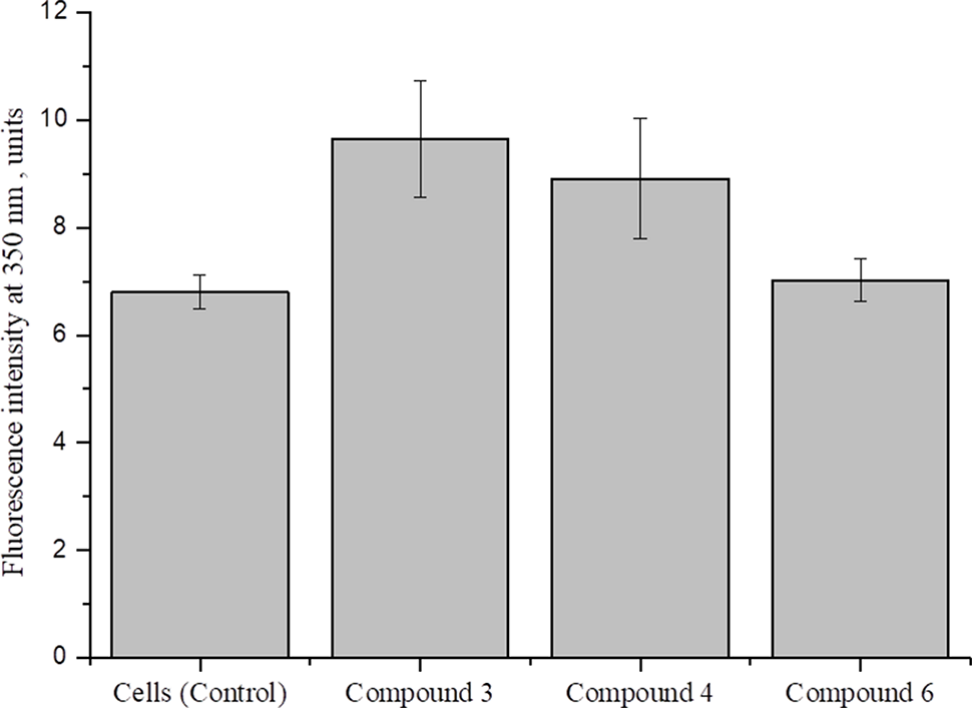
Effect of DNICs on the level of ROS in human lung embryonic fibroblasts.HLEF-104 cells were either kept untreated or treated with DNICs (2 × 10^-4^ M) and intra cellular ROS generation was measured [in terms of peroxide using dichlorofluorescein diacetate (DCF-DA)] as described under *Materials and Methods* section. Data are presented as mean ± SD of three independent experiments. Differences between control and DNICs treated cells are significant at *p ≤ 0.05 with ANOVAs followed by Tukey’s *post hoc* test.

Cellular redox homeostasis is maintained by the balance between ROS generation and successful elimination of ROS by cellular antioxidant capacity (Xiao et al., 2018). It is reported that nitric oxide, generated by high concentrations of DNICs, interacts in mitochondria with superoxide and forms peroxynitrite (ONOO^-^), which is a powerful oxidizing agent (Galijašević, 2013; Scicinski et al., 2015; Ferrer-Sueta et al., 2018). To investigate the involvement of DNICs in oxidative stress, we studied the effect of DNICs on the level of ROS in fibroblasts (**Figure 8**). The results showed that, during the incubation of cells with DNICs, compounds #3 and #4 were not significantly increased the level of ROS, whereas compound #6 had no effect on the level of ROS in fibroblasts. Thus, no significant difference in the level of ROS was observed between control (no DNICs) and low dose of DNICs (2 × 10^-4^ M) samples (P > 0.05).

Therefore, the above results indicate that DNICs don’t affect at the level of ROS at this dose administration.

### Effect of DNICs on ATPase Activity in Fibroblasts Cell Lysates

**Figure 9:**
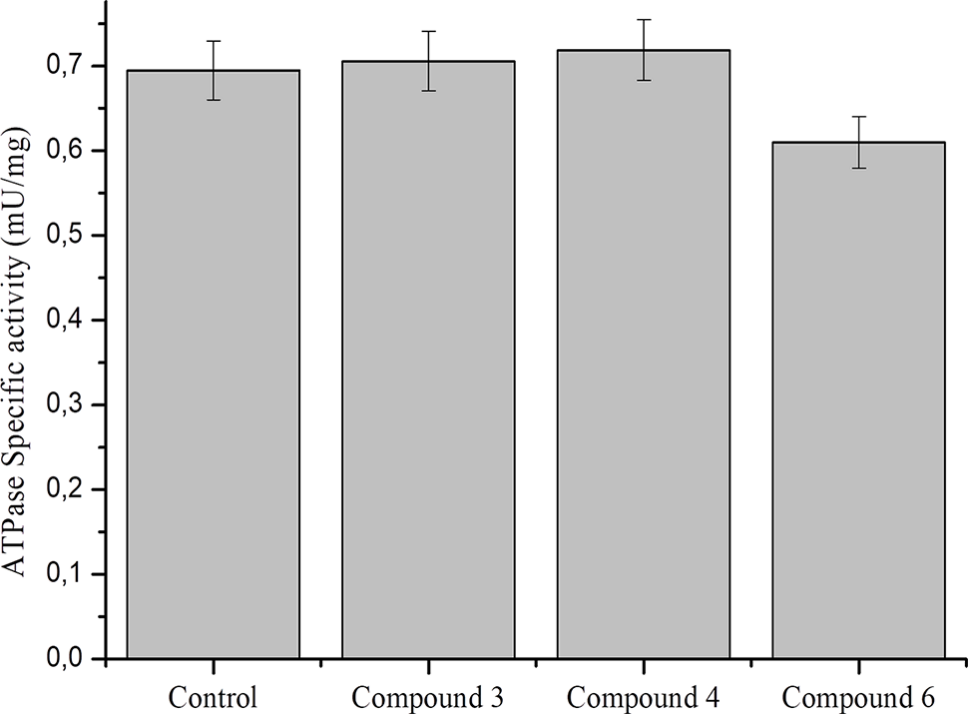
Effect of DNICs at specific ATPase activity in lysates, prepared from fibroblasts. Lysates (1.5 mg/ml) were preincubated 15 min at 4 °C with no additions (1) or in the presence of DNIC#3, DNIC#4 or DNIC#6. Then the ATPase activity was measured as described under *Materials and Methods* section. Data are presented as mean ± SD of three independent experiments. Differences between control and DNICs treated cells are significant at *p ≤ 0.05 with ANOVAs followed by Tukey’s *post hoc* test.

Data on the effect of NO on ATPase activity are inconsistent. It was previously shown that NO donors inhibit the activity of brain and pig kidney Na^+^/K^+^–ATPase (de Lourdes Barriviera, 2005). At the same time, it was demonstrated that NO regulates cardiac intracellular Na^+^ and Ca²^+^ by increasing the activity of Na^+^/K^+^–ATPase (Bogdanova et al., 2016). In particular, NO (spermine-NONO-ate) stimulated Na^+^/K^+^–ATPase *via* phospholemman phosphorylation and thereby limits Na^+^ and Ca^2+^ were overloaded and arrhythmias (Pavlovic et al., 2013; Lu et al., 2015). To further study the role of DNICs, we assessed the effects of DNICs on the level of ATPase activity in fibroblasts. The results revealed that the DNIC#3 slightly increased the level of ATPase activity, and the DNIC#4 and #6 slightly lower the level of activity of this enzyme (**Figure 9**). However, these differences were not statistically significant. Above all, these results showed that DNICs have not affected the level of ATPase in the fibroblasts it remained constant during the processing of DNICs.

### Effect of DNICs on Total ATP in Fibroblasts Cell Lysates

**Figure 10:**
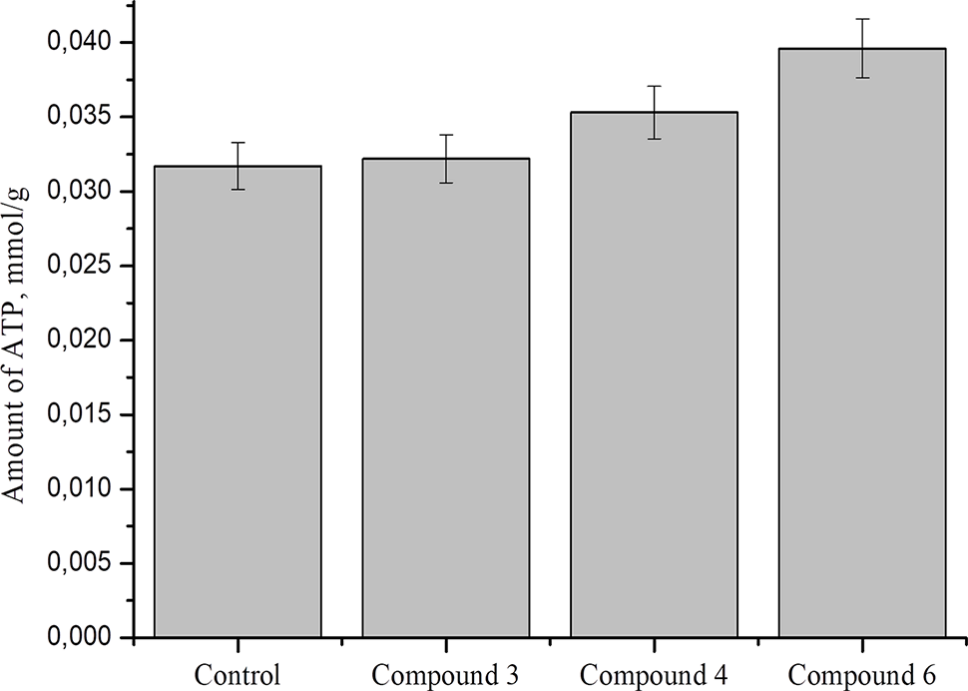
Effect of DNICs on total ATP in fibroblasts cell lysates. HLEF-104 cells treated with DNICs (2 × 10^-4^ M) or untreated (control) and incubated for 15 min at 37 °C, 5% CO_2_. Then prepared cell lysates according to protocol and total ATP was measured as described under *Materials and Methods* section. Data are presented as mean ± SD of three independent experiments. Differences between control and DNICs treated cells are significant at *p ≤ 0.05 with ANOVAs followed by Tukey’s *post hoc* test.

Studies show that nitric oxide reduced ATP level by about 50% in nerve axons and 2016DNICs on the level of total ATP in fibroblasts. The results showed that DNIC#4 and #6 slightly increased the level of ATP in the cell (**Figure 10**). In general, we have established that DNICs does not affect the level of ATP. Taken together, the above results indicated that DNICs maintained the ATP equilibrium in cell.

## Discussion

Donors of NO are widely used drugs for the treatment of cardiovascular diseases (Katsumi et al., 2007; Ahmad et al., 2018). It is known that NO ensures the normal functioning of the cardiovascular system in physiological conditions and its adaptation in conditions of pathology (Bian et al., 2008; Divakaran and Loscalzo, 2017). Development of NO delivery systems, i.e., the synthesis of new representatives of low molecular weight NO donors (nitrates, nitrites, nitramines, 1,2-diazeta-1,2-dioxides, guanidines, furoxanes, oximes, sydnonimines, diazeniumdiolates (NONOates), S-nitrosothiols, nitrosyl metal complexes) and the development of macromolecular NO-releasing molecules (dendrimers, polymers/films, particle-type and coating platforms for NO delivery) is becoming increasingly important in biomedical applications of NO (Granik and Grigor’ev, 2002; Carpenter and Schoenfisch, 2012; Kim et al., 2014; Sanina et al., 2015). Most of the available low molecular NO donors have essential drawbacks: organic and inorganic nitrates induce nitrate tolerance; sydnonimines yield superoxide anions together with NO release, thus forming carcinogenic peroxynitrite (ONOO^-^) and giving rise to pathogenic conditions *in vivo*; wide application of NONO-ates, effective class of organic NO donors, is limited by their high cost; S-nitrosothiols are unstable upon storing and are cytotoxic because in the presence of redox agents they form thiol radicals recombining quickly to form disulfides and nitrosonium ions, which hydrolyze to yield nitrite anions. Thus, with the exception of cyanonitrosyl metalates [M(CN)_x_NO_y_]^n^, which might accumulate cyanides upon decomposition, most of the available compounds and pharmaceutical substances are able to control the NO amount *in vivo* are synthetic organic compounds (McCleverty 1979; Darius et al., 1984; Torfgard and Ahlner, 1994; Keefer et al., 1996; Rosenkranz et al., 1996; Anthony and Rhodes, 1997; Blackburn et al., 1998; Oszajca et al.,1998; Gaston 1999; Fitzhugh and Keefer, 2000; Lee et al., 2001; Wöhrle et al., 2003; Morphy et al., 2004; Sanina et al., 2015). However, the discovery of the role of intracellular nitrosyl non-heme proteins in the biochemistry of NO has led to the rapid development of a new line of research based on the use of synthetic analogues of these highly reactive nitrosyl cell intermediates as prodrugs that release NO (Constanzo et al., 2001; Butler and Megson, 2002; Yang et al., 2002; Turella et al., 2003; Tsai et al., 2004; Lancaster 2010; Islam 2015; Sanina et al., 2015). These complexes are models of the active sites of nitrosyl non-heme [Fe-S] proteins existing in all living organisms, from bacteria to mammals (Butler and Megson, 2002; Lewandowska et al., 2011; Sanina et al., 2015). These mono- and binuclear nitrosyl iron complexes with functional sulfur-containing ligands have some advantages over other low-molecular NO-donating agents: these compounds release NO at physiological pH values without any activation (photo, thermal or redox), they can be isolated in the crystalline state, and no toxic products form upon their decomposition, this being favorable for biological and medical investigations and applications (Sanina et al., 2015). Recently it was obtained and characterized a new family of cationic water soluble dinytrosyl iron complexes (DNICs) with S-donor ligands, such as thiocarbamide and its derivatives (Sanina et al., 2014a; Sanina et al., 2015; Sanina et al., 2016; Shmatko et al., 2017a). These compounds have several advantages over previously synthesized NO donors. Firstly, they are water-soluble compounds. Secondly, it was shown that these DNICs are more effective NO donors compared to the commercial NO donor diethylenetriamine and have more than twice the ability to generate NO (Sanina et al., 2014b; Sanina et al., 2015). Their structure and properties has been studied previously by X-ray diffraction analysis, IR-, Mössbauer- and EPR spectroscopy (**Figure 1, Supplement Material**) (Shmatko et al., 2017a). DNICs serve as carriers of the “ready-to-use”, iron-stabilized form of NO (Kapelko et al., 2017; Schiewer et al., 2019). It was shown that the mechanism of NO release from iron–sulfur complexes in aqueous solution is similar to the generation of NO in the solutions of monocation iron complex with thiocarbamide [Fe(SC(NH_2_)_2_)_2_(NO)_2_]ClH_2_O (Sanina et al., 2014a; Sanina et al., 2015). This mechanism involves the dissociation of the Fe–NO bond and the replacement of NO by the aqua ligand by adding water to the free coordination center of iron (Sanina et al., 2015). Apparently, stable nitrosyl intermediates formed during the decomposition of DNICs are present in solutions, which provide long-term NO-donor properties of the complexes (Sanina et al., 2015). The effect of various DNICs depends on their chemical and electronic structure, the reactivity of nitrosyl groups, amount of NO generated and the duration of its release.

Depending on this, some DNICs exhibit cytotoxic properties and are potential antitumor drugs but other DNICs exhibit cytotropic, cytoprotective and vasodilation properties and therefore are potential drugs for the treatment of cardiac diseases and cardiac complications after chemotherapy.

As follows from a comparative analysis of the molecular and crystalline structure of DNICs compounds, the conformation of the [Fe (SR)_2_ (NO)_2_]^2+^ cation changes depending on the nature of the counter ions in the structure, which is due to the rotation of thiocarbamide ligands around Fe–S and S–C bonds In addition, the type of intermolecular interaction in salts of [Fe (SR)_2_ (NO)_2_]^2+^ with various counter ions affects the structure of thiocarbamide ligands as well as the structure and reactivity of nitrosyl groups (Sanina et al., 2015). General formula of cationic mononuclear DNICs is [Fe (SR)_2_ (NO)_2_] ^n^^+^ X^-^, where n = 1-4, X = acid residue, R represents thiourea, as well as its derivatives by the reaction of aqueous solutions of iron salts (Aldoshin et al., 2016). However, the molecular mechanism of DNICs action and their metabolic effects in cells has not been sufficiently studied. In this paper we first studied the metabolic effects of DNICs at human fibroblasts and rat cardiomyocytes. We proceeded from the fact that earlier, for other NO donors, cytoprotective and cardioprotective effects were shown at their low concentrations (<1 µmol) (Davis, et al., 2001; Naseem, 2005; Lewandowska et al., 2011; Xia and Vanhoutte, 2011; Omar et al., 2016; Komatsu et al., 2018; Hou et al., 2019; Raffetto et al., 2019; Taysi et al., 2019; Tegeder, 2019). It was previously established that DNICs (0.4 × 10^-5^ M) decompose to form NO in the first second after dissolution in an anaerobic aqueous solution (**Figures 2**–**4, Supplement Material**) (Sanina et al., 2013; Sanina et al., 2014a). In particular, the maximum amount of NO generated by DNIC # 3 at pH 7 was ∼ 16 nM, complex 4 was 8 nM, complex # 6 was 2.5 nM after 50 s (**Figures 2**–**4, Supplement Material**) (Sanina et al., 2014). Based on this we weighed (2 × 10^-4^ M) of DNICs and then checked the generation of NO using the electrochemical method (**Figure 1**). According to these data, DNICs were generated from 8 to 16 nmol of NO. In particular, DNIC#3 at pH 7 generated∼ 16 nM of NO, and DNIC#4 released 8 nM of NO after 50 s. Thus, these values are consistent with previously published data on the release of NO (Sanina et al., 2014a). Some threshold concentration of the cytotoxicity effect of NO has been demonstrated previously (Liu et al., 2019). The threshold doses for NO^•^ induced cytotoxicity were shown to be 150 µM min in TK6 cells and 300 µM min in NH32 cells, respectively. However, in our work, DNICs were generated from 8 to 16 nmol of nitric oxide (this amount NO is lower in 20,000 times compare with amount of NO, used by Li et al.: 150 and 300 µM). As mentioned above NO donors demonstrated cytotropic and cardioprotective effects at low concentrations (<1 µmol).Therefore, we chose the initial concentration DNICs (2 × 10^-4^ M), which, when dissolved, released 8 to 16 nmol of NO.

Furthermore in this study, our findings suggested that DNICs were not only non-toxic, but even exhibited cytotropic properties in fibroblasts and rat cardiomyocytes. We studied the effect of DNICs on cell viability for a short and long time. It was found that the effect of DNICs on cell viability manifests itself after 15 min, since an increase in fluorescence intensity is observed. However, the maximum effect was observed after 10 h. Then, the cell viability decreased, however, even 30 h later it remained 3–5 times higher than in the control cells, which were not treated with DNICs. We believe that DNICs increased the activity of mitochondrial NADH dehydrogenase, metabolic processes occur more intensively under the influence of DNICs and thereby increase the cell viability. These results indicate that DNICs are cytotropic agents. It was previously shown that NO able to trigger mitochondrial biogenesis in cells as diverse as brown adipocytes and 3T3-L1, U937, and HeLa cells (Nisoli et al., 2003; Lascaratos et al., 2012). However, the authors indicated that this effect of NO was dependent on guanosine 3′, 5′-monophosphate (cGMP) and was mediated by the induction of peroxisome proliferator-activated receptor γ coactivator 1α, a master regulator of mitochondrial biogenesis. It is possible that in our case, DNICs also affect the mitochondrial biogenesis, however, their mechanism of action is different. Nisoli et al. showed that the NO–cGMP-dependent pathway controls mitochondrial biogenesis and body energy balance. In our work, we showed that DNICs increased the activity of mitochondrial dehydogenases, however, did not affect the level of ATP. We suppose, that treatment with DNICs led to enhancement of the activity of mitochondrial dehydrogenases, and by this prevent cells from induction of apoptosis. We observed the cells under a microscope before measuring viability; cells looked attached to the surface and spread out. These cells are adhesive, and if apoptosis occurred, they would round off, detach from the surface and begin to swim in a growth medium. The number of cells did not change under the influence of DNICs and we did not observe an increase in the number of apoptotic cells either.

We also studied the cytoprotective effect of DNICs against toxicity induced by doxorubicin, anti-tumor drug. It is well-known that doxorubicin is highly toxic and causes many complications in the cardiovascular system after chemotherapy treatment. It had been reported, that doxorubicin is antibiotic of the anthracycline series and has antimitotic and anti-proliferative effects on cells (Rivankar, 2014; Dubbelboer et al., 2017). It is known in the literature that doxorubicin induces oxidative stress by ROS overproduction and accordingly reduces the level of glutathione in the cell, diminishes ROS-scavenging activities (e.g. catalase and glutathione peroxidase), significantly decrease the inner mitochondrial membrane potential and ATP level (Serafino et al., 2000; Childs et al., 2002; Mukhopadhyay et al., 2009; Kuznetsov et al., 2011; Sifaoui et al., 2014). This manifests the cytotoxic effect of doxorubicin. Doxorubicin binds to DNA in mitochondria, destroys it, decrease the membrane potential, which leads to an increase in ROS and induction of apoptosis, followed by cell death. The principle of using doxorubicin in oncology is based on this. We included doxorubicin as a control to compare its toxic effect on cell viability with the protective effect of DNICs and to demonstrate a decrease in its toxicity due to preliminary incubation of cells with DNICs. Results showed that doxorubicin caused a decrease in the cell viability, whereas the cells that were treated with compounds #3, 4, and 6 retained a higher viability: their survival was two- to three times higher than that of the untreated cells. DNICs#3 and #4 have been shown to be the most effective cytoprotectors. Therefore, our findings demonstrate that DNICs are effective cytoprotectors with a long-term effect, which protect cells from the toxic action of doxorubicin.We also studied the effect of DNICs at the mitochondrial membrane potential on cells. Our data shown that incubation cells with DNICs decreased mitochondrial transmembrane potential (ΔΨ_m_) not significantly, just in 2–3 times. Results demonstrated that effect of DNICs on membrane potential was soft, moderate and, probably, short-term and reverse. It was previously shown that long-term treatment with doxorubicin also resulted in a quick drop in membrane potential (in 5 times), followed by detachment up to 90% cells and cell death (Kuznetsov et al., 2011). In our case, we believe that this short-term drop in the membrane potential is useful for the cell, as it allows cells to get rid of excess charge and thereby reduce the formation of ROS, prevent apoptosis and protect cells from death. In addition, we suppose that slight depolarization of the mitochondrial membrane is short-term and reversible, and after a while the normal ΔΨ_m_ is restored. When setting up the experiments, we also observed the state of the cells under a microscope. These cells are adhesive, in a normal state they are spread over the surface. Microscopic analysis of the cells showed that after preliminary treatment of the cells with DNIC and subsequent treatment with doxorubicin, the cells did not round up, they remained attached to the surface, indicating their normal viability.

It’s obvious that the mechanism of DNICs action on the membrane potential is different from the mechanism of action of doxorubicin. It has previously been reported that mechanism of action of doxorubicin includes the cooperation with DNA, generation of free radicals, and direct effect on cell membranes with the suppression of the synthesis of nucleic acids (Dubbelboer et al., 2017). Therefore, the treatment of cancer with doxorubicin is accompanied by an increase in the production of free radicals in the mitochondria, which have a deleterious effect on the structure of both tumor and normal cells as a result of oxidative stress (Cooley, 2007). This is accompanied by numerous side effects of doxorubicin, which are toxic to the whole body during the chemotherapy. For example, the cardiovascular system disorders include cardiomyopathy, heart failure, and arrhythmia (Koleini and Kardami, 2017). This process directly depends on the mitochondrial membrane potential: the higher the latter, the greater amounts of free radicals are produced by the mitochondria. Thus, most adverse effects of doxorubicin on the normal cells are associated with the oxidative stress. However, this oxidative stress can be reduced by providing the leakage of excess charge through the mitochondrial membrane (Wang et al., 2009, Montaigne et al., 2012; Islam, 2017). This protective mechanism is called the uncoupling of oxidative phosphorylation. It is used by cells *in vivo* but can also be induced by synthetic compounds. Natural mechanisms of uncoupling do not always cope with their functions. It has been shown that reduction in mitochondria membrane potential is often seen in pathological conditions. Many pathological conditions have been described in which mitochondrial hyperpolarization occurs, accompanied by an increased level of ROS in the mitochondria, which leads to a deterioration in the functioning of the cell as a whole. This situation occurs in obesity, impaired carbohydrate metabolism, as well as in pathologies associated with the development of oxidative stress: ischemic tissue damage, Parkinson’s and Alzheimer’s disease, autoimmune diseases (Skulachev, 1996; Silachev et al., 2018). The use of uncouplers capable of regulating by increasing the proton conductivity of mitochondria is capable of eliminating the negative effects caused by these pathological conditions, in particular, by controlling the level of ROS generation by mitochondria (Korshunov et al., 1997; Rolo and Palmeira, 2006). The vast majority of uncouplers are negatively charged molecules, anions, the uncoupling mechanism of which is similar to fatty acid anions (RCOO^-^). It was found that certain chemical agents selectively accumulate in mitochondria and reduce their membrane potential and, thus, exhibit cytoprotective properties (Normoyle et al., 2015; Kanemoto et al., 2019; Khailova et al., 2019). These compounds can be used to prevent severe neurological disorders after ischemic stroke (Kwok et al., 2010; Perez-Pinzon et al., 2012; Normoyle et al., 2015). However, the possibility of medical use of such molecules is limited to an extremely small therapeutic window—the difference between therapeutic and toxic doses of the drug. The fact is that a sharp decrease in the membrane potential leads to a disruption in the main function of the mitochondria–ATP synthesis, the universal “fuel” necessary to maintain the vital activity of the cell and the organism as a whole (Nabben et al., 2011).

Currently, a group of academician Skulachev has created a number of substances—cationic uncouplers, whose chemical structure allows them to selectively accumulate in mitochondria and reduce excess mitochondrial potential (Lyamzaev et al., 2018; Silachev et al., 2018; Genrikhs et al., 2019). At the same time, the effect of cationic uncouplers is self-regulating—since these are negatively charged molecules, the rate of their penetration into the mitochondria is directly proportional to the mitochondrial potential. However, the mitochondrial potential decreases with increasing concentration of the cationic uncoupler inside the mitochondria. It was shown that the new cationic uncoupler—rhodamine-19 butyl ether (C4R1) effectively accumulates in mitochondria to a certain concentration, without disturbing their vital functions. In experiments on rats—models of ischemic stroke, it was shown that the introduction of C4R1 after a reversible violation of cerebral circulation significantly reduced the scale of brain damage to animals and related neurological disorders (Korshunova et al., 2017; Lyamzaev et al., 2018; Silachev et al., 2018; Genrikhs et al., 2019). It should be noted that preventing the production of oxygen radicals in mitochondria is a fundamental principle in the fight against many diseases. Such compounds will have the properties of an uncoupler that normalizes mitochondrial potential. Clinical studies on the use of such molecules in the treatment of a number of diseases are being actively conducted. Finally, nitroglycerin has been shown to induce a protective phenotype that limits damage after ischemia and reperfusion. Nitroglycerin protects against post-ischemic endothelial dysfunction in particular, in part, by impairing the opening of the mitochondrial permeability transition pore (Gori et al., 2008; Divakaran and Loscalzo, 2017).

We assume that DNICs may uncouple of oxidative phosphorylation in the cell and thereby lower the mitochondrial membrane potential (Aly and Domenech, 2009). This assumption is based on the structure of DNICs. The total DNICs formula ([Fe (SR)_2_ (NO)_2_] ^n^^+^ X^-)^ corresponds to the general formula of cationic uncouplers: (X–Z)^n+^A^n-^, where “X” is a group containing heteroatoms such as –S^-^, –N^-^, –O^-^; “Z” is a group that can protonate at physiological pH values and provide targeted delivery of the entire compound to mitochondria; “A” is the counterion; “N” is an integer from 1 to 3, as well as its tautomeric forms, solvates, salts, isomers or prodrugs, and a pharmacologically acceptable carrier. In addition, DNICs have negative charge and they can penetrate through membranes. We suppose, that tested DNICs have a double mechanism of action, in particular, they exhibit both the ROS-scavenging effect and mitochondrial effect. DNICs include two parts: NO donor and [Fe–S] cluster (mitochondrial targeting). NO is high reactivity because of its electronic configuration, i.e., existence of an unpaired electron on the π-molecular orbital (Wong et al., 1989). According to this released NO can interact with radicals (O^2-^, H^+^) and prevent the oxidative stress, working as antioxidant. The iron in the [Fe–S] cluster is monovalent and therefore it can interact with protons and pass into the divalent or trivalent state. Therefore, the [Fe–S] cluster can work as soft uncoupler and cause a drop in the mitochondrial potential. In addition, high affinity of NO for Fe^2+^ results in the interaction of nitric oxide with a variety of iron-containing proteins, for example, ribonucleotide reductase, ferritin, [Fe–S]clusters of NADH-reductase, NADH dehydrogenase (Rahmanto et al., 2012; Shmatko et al., 2017b). It is known that NADH-dehydrogenase complex, also called complex I is the first multi-protein complex of the electron transport respiratory chain. In relation to human proteins, complex I is often called NADH-dehydrogenase (Birrell et al., 2009). Many copies of the complex are located in the inner mitochondrial membranes of eukaryotic cells. NADH-dehydrogenase complex consist from one flavin mononucleotide and from 8 to 9 iron-sulfur clusters (prosthetic groups) (Voet and Voet, 2004). This complex plays a central role in cellular respiration and oxidative phosphorylation: almost 40% of the proton gradient for the synthesis of ATP is created precisely by this complex (Efremov et al., 2010). Moreover, it is own that NO inhibits the mitochondrial respiratory pathways (Montaigne et al., 2012). We suppose that DNICs generate the NO, which interacts with [Fe–S] clusters of NADH–dehydrogenenase, change its conformational state and enhances the activity of NADH–dehydrogenase. In addition, the ligands, remaining after generation of nitric oxide, [Fe–S] clusters can be used as prosthetic groups of NADH dehydrogenase and thereby increase its activity. NADH–dehydrogenase complex oxidizes NADH, which is formed in the matrix during the tricarboxylic acid cycle (Berg et al., 2006). Electrons from NADH are used to restore the membrane carrier, ubiquinone Q, which transfers them to the next complex of the electron transport chain of mitochondria, complex III, or the cytochrome bc1 complex (Berg et al., 2006). As a result of DNICs effect, NADH-dehydrogenases break down a larger amount of NADH and a large number of protons (electrons) are formed, which is used in various metabolic and energy processes in the cell. As a result, cell viability increases. However, the increased activity of dehydrogenases after a certain time leads to the accumulation of excess charge in the mitochondria (Murphy, 2009). Excessive number of electrons can lead to an increase in the formation of ROS, oxidative stress and, as a result, cell death (Murphy, 2009). We suggest that DNICs (in particular, NO and Fe–S clusters) are also able to attract electrons to themselves, which leads to the removal of excess charge through the mitochondrial membrane. The consequence of this is the uncoupling of oxidative phosphorylation, accompanied by a decrease in ROS, a decrease in the mitochondrial membrane potential and a suspension of ATP synthesis (Yang T. et al., 2018). This is a protective effect of DNICs. However, it is likely that the decrease in membrane potential is insignificant and short-term, since a sharp decrease in membrane potential leads to disruption of ATP synthesis. After some time, the membrane potential returns to its normal state. It is known that the reaction catalyzed by complex I is reversible, this process is called aerobic succinate-induced reduction of NAD ^+^ (Grivennikova et al., 2007). Under conditions of high potential on the membrane and an excess of reduced ubiquinols, the complex can reduce NAD ^+^ using their electrons and pass protons back into the matrix (Kwok et al., 2010).

**Figure 11:**
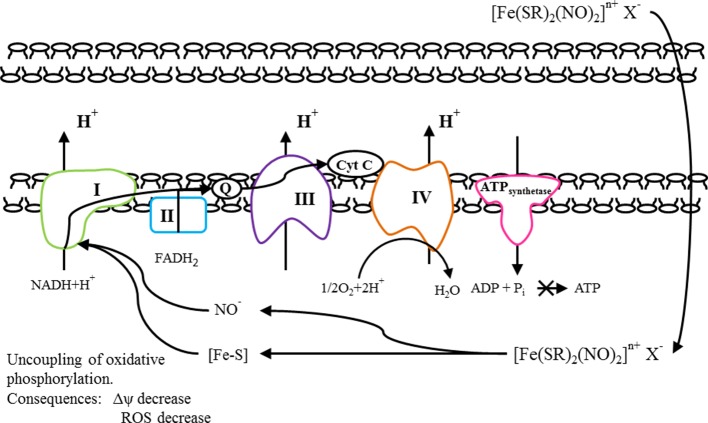
The proposed mechanism of action of DNICs.

The proposed mechanism of action of DNICs is shown in **Figure 11**. All this together determines their cytotropic and cytoprotective properties.

We investigated the effect of DNICs on cell proliferation and, in parallel, counted the number of living and dead cells before flow cytometry analysis. The results showed that the number of dead cells increased slightly after treatment with DNIC by only 1–2%. Flow cytometry analysis showed that DNICs did not affect cell proliferation. However, we observed an obvious increase in the subG1 phase in cells treated with compounds 3 and 4. We suggest that such a small increase in apoptotic cells occurs as a result of mechanical stress, which often occurs in cells by excessive pipetting, shaking, or rapid centrifugation >350g during cell fixation, resulting in subG1 fragments that occur during harvesting and staining.

It has previously been reported that ATPase (Adenosine Triphosphatase: EC 3.6.1.3) is an important enzyme for maintaining the cell membrane potential, transporting ions and regulating cellular volume (Riley and Peters, 1981). We therefore tested the effect of DNICs on the ATPase enzyme activity and the total amount of ATP in the cells. Our results showed that DNICs did not affect the level of ATPase and the total amount of ATP in the cell, its level remained constant. Therefore, our findings suggested that DNICs are not involved in the signaling pathway for the synthesis and breakdown of ATP in the cell.

It was previously found that maintaining the optimal ratio of reduced glutathione (GSH) to oxidized (GSSG)—GSH/GSSG is an important condition for cell viability (Kim et al., 2010; Bachhawat and Yadav, 2018; Parvez et al., 2018). Glutathione plays a key role in redox regulation of the main processes of cell activity, namely, in regulation of the cell cycle, energy production, apoptosis, proliferation, differentiation, protein folding, transcription, DNA repair, signaling, and antioxidant protection (Traverso et al., 2013; Lu et al., 2016; Robaczewska et al., 2016; Zhang et al., 2018). Glutathione is a low molecular weight antioxidant and can participate in non-enzymatic antioxidant protection, acting as an effective scavenger (trap) of free radicals (Forman, 2016). A decrease in the GSH level below normal values serves as an indicator of a violation of the cellular redox status and changes in the redox-dependent regulation of genes (Rushworth and Megson, 2014; Forman, 2016). Thus, we investigated that role of DNICs on the level of glutathione in cells. The finding of the study showed that the tested DNICs did not affect the level of reduced glutathione in the cells. Moreover, this indicates that DNICs does not cause oxidative stress in cells and the redox balance is not disturbed. Recent studies have shown that GSH accumulates in the nucleus at the beginning of the G1 phase, so it can play an important role in maintaining the redox status of the nucleus during the cell cycle (Garcia-Gimenez et al., 2013). In addition, studies have shown that cancer cells have an elevated level of glutathione, which allows them to quickly divide and be resistant to oxidative stress (Estrela et al., 2016). Since the level of glutathione can influence cell proliferation, we investigated the effect of DNICs on cell division using flow cytometry. We found that DNICs were not affected cell proliferation. Taken together the results suggested that since DNICs were not affected on the level of glutathione, it retains the redox status of the nucleus during the cell cycle and the level of cell proliferation was not changed.

Reports show that under normal conditions, the GSH/GSSG ratio is 100:1. Maintaining an optimal GSH/GSSG ratio in the cell is essential for its normal functioning and survival (Tchouagué et al., 2019). Violation of this ratio has a significant impact on the processes of signal transduction, control of gene expression, cell proliferation, differentiation, the state of cellular metabolism, and cell activity in general (Nagapan et al., 2019). The lack of GSH exposes the cell to the risk of oxidative damage and leads to the accumulation of ROS in the cell. ROS play an important role of signaling molecules; however, their accumulation in pathological states leads to oxidative stress (Sarniak et al., 2016; Ravera et al., 2015). The main source of ROS in cells is oxidative phosphorylation. Mitochondrial dysfunction and oxidative stress are involved in the pathogenesis of many diseases (Grancara et al., 2015; Liu et al., 2019). Oxidative stress causes many degenerative diseases and cell death. The formation of reactive oxygen species with the participation of mitochondria plays an important role in the induction of apoptosis in pathophysiological processes in neurons, cardiomyocytes, and also in the process of aging (Haller et al., 2002; Czarna and Jarmuszkiewicz, 2006; Lin et al., 2012; Lin et al., 2012; Kleme et al., 2018; Salimi and Pourahmad, 2018; Suski et al., 2018). Therefore, the determination of ROS can provide important information about the physiological state of the cell and the function of mitochondria (Liu et al., 2019). Based on these results, we investigated the role DNICs in formation of ROS. As expected, this study showed that showed that DNICs at this dose of administration (2.0 × 10^-4^ M) were not affected the level of ROS in the cell, that is, peroxynitrite (ONOO^-^) was not formed in the cell, which has a high oxidative activity. It is possible that the DNICs protected cells (fibroblasts and rat cardiomyocytes) from ROS by maintaining redox balance. This finding indicated that DNICs caused no physiological disorders in fibroblasts and rat cardiomyocytes. It is possible that DNICs prevent the production of oxygen radicals in the mitochondria, which is a fundamental principle in the fight against many diseases.

For the first time we found that DNICs were protective against doxorubicin-induced toxicity in fibroblasts. Therefore, our findings suggested that DNICs might protect cells *in vitro* against doxorubicin, a known anti-tumor and cardiac toxic anthracycline antibiotic. We suggest that DNICs can briefly uncouple the electron transport chain and thereby induce the leakage of excess of electrons through the membrane and prevent the occurrence of oxidative stress in the cell. We have demonstrated here that these DNICs can be used in cardio-oncology to maintain or increase the viability of cells of the whole body and cardiomyocytes in the treatment of cancer.

## Conclusion

Above all, the study showed that the above mentioned results revealed that DNICs have the cytotropic and cytoprotective effects on cells.

Moreover, we are first demonstrated that DNICs can be regarded as cationic mitochondrial uncouplers. These compounds did not disturb the vital metabolic processes in the cell, but, on the contrary, demonstrated a protective effect and increased the cell viability of fibroblasts and rat cardiomyocytes.

This indicates that DNICs are promising compounds for the treatment of cardiovascular diseases (ischemia and stroke) and cardiac complications in the treatment of cancer patients. Assessing the data obtained from a practical point of view, it can be concluded that DNICs are promising candidates for further pharmacological studies to develop drugs for the treatment of CVD. However, further research is needed on preclinical and clinical trials of these compounds.

## Data Availability Statement

The raw data supporting the conclusions of this manuscript will be made available by the authors, without undue reservation, to any qualified researcher.

## Author Contributions

NA contributed conception, designed and performed experiments, analyzed and interpreted of data for the work, wrote the article. NAS designed and synthesized DNICs. AG performed experiments, wrote sections of the manuscript. NIS organized the database, performed experiments, designed sections of the article. TP performed the statistical analysis. SS contributed design of experiments, revised work critically for important intellectual content. NZ contributed the acquisition, analysis and interpretation of data for the work. SA contributed conception of the study and provided approval for publication of the content. All authors contributed to manuscript revision, read and approved the submitted version.

## Funding

The work was supported by the Fundamental Research Program N 1.42 P “Fundamental Research for Biomedical Technologies” 2018 – 2019 year.

The work was carried out with the financial support of the FASE, the number of the state registration of research: # 0089-2019-0014.

## Conflict of Interest

The authors declare that the research was conducted in the absence of any commercial or financial relationships that could be construed as a potential conflict of interest.
