# Supplementary figures and images for: Cytoprotective Effects of Dinitrosyl Iron Complexes on Viability of Human Fibroblasts and Cardiomyocytes
Source: Front Pharmacol. 2019 Nov 11;10:1277. doi: 10.3389/fphar.2019.01277 (PMC6859909; doi:10.3389/fphar.2019.01277)

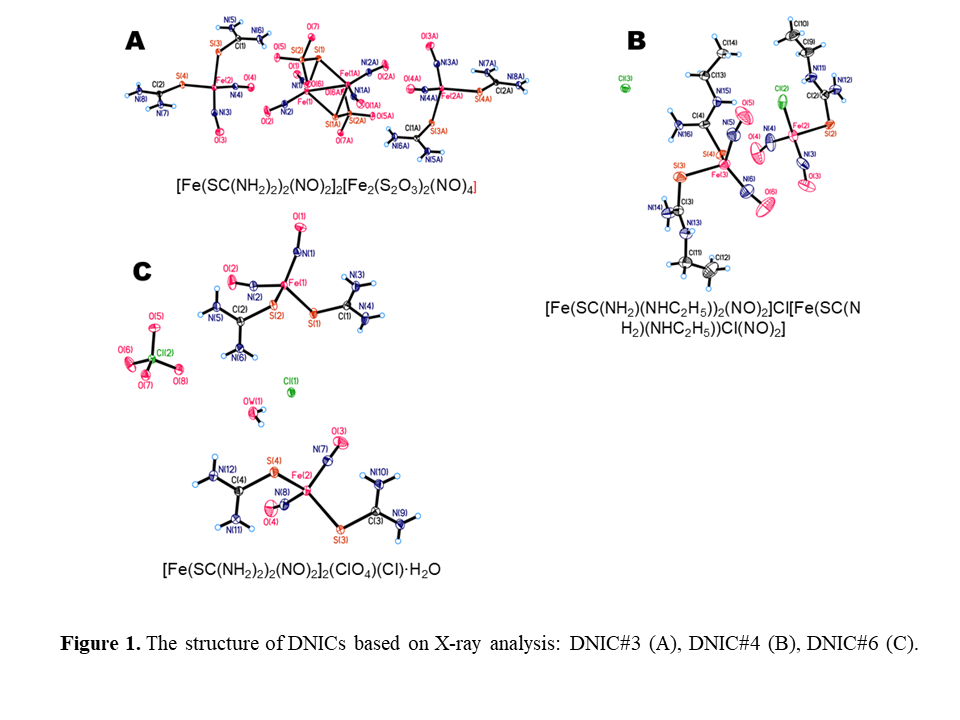

Supplement: Supplementary file 1 [file Image_1.tif]

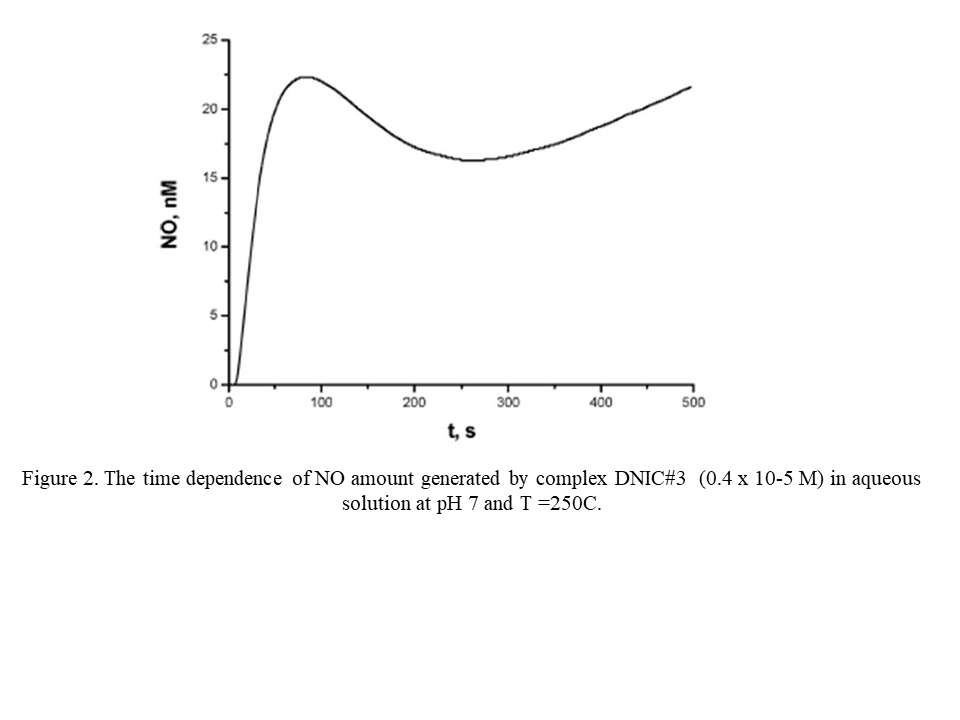

Supplement: Supplementary file 2 [file Image_2.tif]

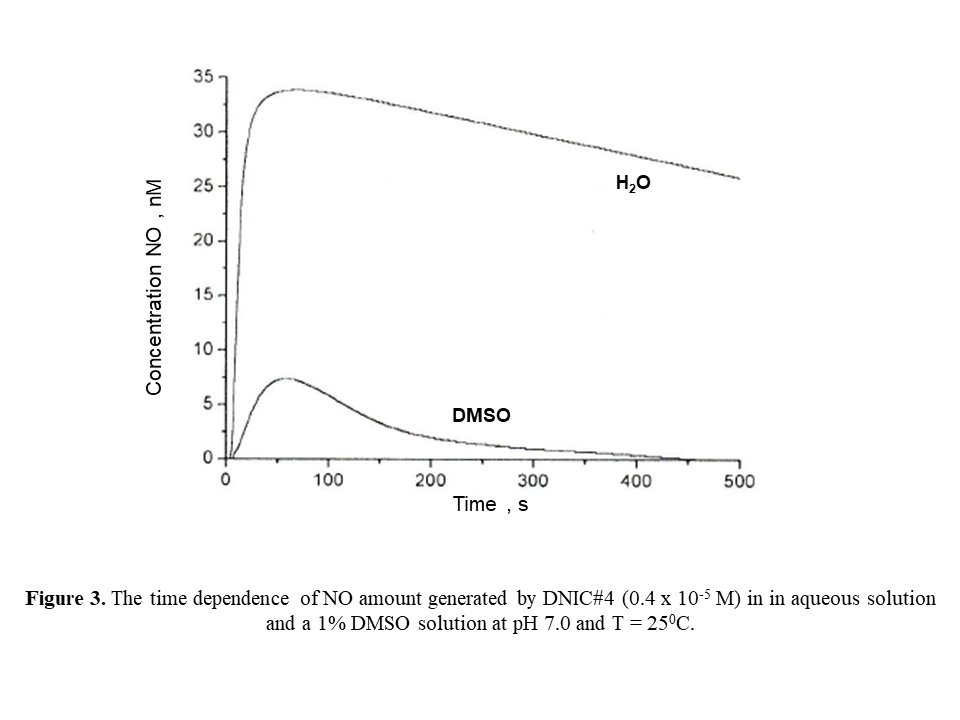

Supplement: Supplementary file 3 [file Image_3.tif]

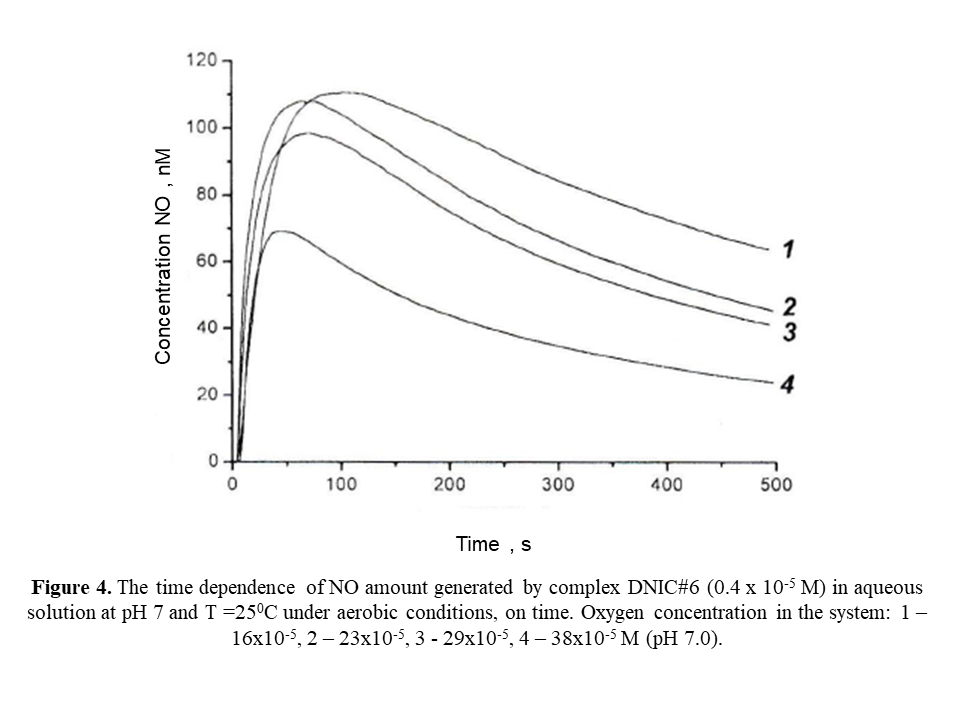

Supplement: Supplementary file 4 [file Image_4.tif]
